# Behind the wall: Macrolithic artifacts as testing tools for activities and social structure on a Middle Chalcolithic site in Central Anatolia

**DOI:** 10.1371/journal.pone.0319698

**Published:** 2025-04-14

**Authors:** Jaroslav Řídký, Kristina Doležalová, Daniel Pilař, Pınar Çaylı, Işıl Demirtaş, Sevil Gülçur

**Affiliations:** 1 Institute of Archaeology of the Czech Academy of Sciences, Prague, Czech Republic; 2 Institute of Classical Archaeology, Faculty of Arts, Charles University, Prague, Czech Republic; 3 Department of Archaeology, Nevşehir Hacı Bektaş Veli University, Nevşehir, Türkiye; 4 Department of Archaeology, Aksaray University, Aksaray, Türkiye; 5 Prehistory Department, Faculty of Letters, Istanbul University, Istanbul, Türkiye; Universidad de Sevilla, SPAIN

## Abstract

The main focus of this work is an assemblage of nearly two thousand macrolithic artifacts, weighing more than half a metric ton, retrieved from an excavated area measuring 3,600 m^2^ of the Middle Chalcolithic site of Güvercinkayası, which dates from the end of the 6^th^ and first half of the 5^th^ millennium cal BCE. The site is ideal for testing different analytical approaches due to the extent of the excavated area, the good preservation of structures and finds, the site layout and the fact that the site is divided into two parts by a fortification wall. Macrolithic artifacts were divided into ten classes according to the method of manufacture, raw materials, shapes and dimensions, and their functional use. Several examples were chosen for use-wear analysis of the active surfaces. The Middle Chalcolithic period in Central Anatolia represents the end of previous Neolithic traditions in architecture and ways of life. Therefore, the main objective of our work is to present the assemblage from this period, and further to test the information potential of these artifacts for studying the activities and social structure of settlements. The shape spectrum of the assemblage largely corresponds to the Neolithic period. It differs mainly in the proportion of some shapes and functional types present. In addition, certain artifacts appear to indicate the existence of some form of counting system or board game. Based on the results of the study of macrolithic artifacts and their statistical and spatial analysis, it is clear that the composition of artifacts and access to raw materials was similar in different parts of the settlement. The inhabitants of the Middle Chalcolithic site created a settlement with a regular layout, which at a certain time was divided into two parts. However, the results of our analyses do not demonstrate any significant patterns that would testify to vertical social stratification of the society living within this settlement.

## Introduction

**Fig 1:**
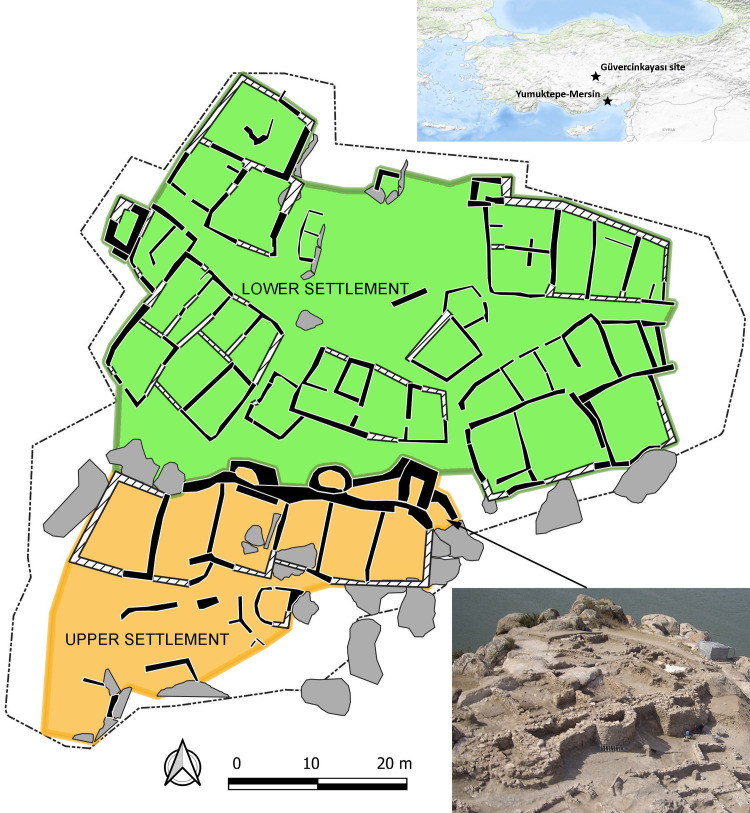
Location and general plan of the MCH site of Güvercinkayası in Central Anatolia with a fortification system dividing the area into lower and upper settlements. In black: Ground plans of excavated houses; in grey: rocks. Plan by D. Pilař; photo GK archive; background map: US Geological Survey (USGS).

One of the most durable materials from which artifacts are made is stone. Although different categories of stone artifacts have been studied for many decades, their informational potential is still far from exhausted (e.g., [1]). In this work we will focus on two issues associated with so-called macrolithic artifacts ([2]; the term ground stones is also used; e.g., [3]). In the first part we present an extensive assemblage of this find category from the Middle Chalcolithic site of Güvercinkayası in Central Anatolia (Fig 1; S1 Video; west Cappadocia; 5,200 – 4,820/4,750 cal BCE; [4,5]). While studies have been undertaken of this find category from the preceding Neolithic period in today´s Türkiye and surrounding countries [6–12], a more detailed overview for the Chalcolithic, especially for the dynamic Middle Chalcolithic period (henceforth abbreviated as MCH; [13–15]), is still lacking (e.g., [16,17]). In the past, a substantial portion of macrolithic artifacts (henceforth abbreviated as MA) generally served for various food processing activities and for a variety of craft technologies (e.g., [11,18–21]), which, in better preserved contexts, often appear to be located in specific activity areas of the settlements (e.g., [6,22–27]). Subsequently, the second and most important part of the work will focus on MA distribution patterns evident in the archaeological record of the spatially organized Güvercinkayası site.

**Fig 2:**
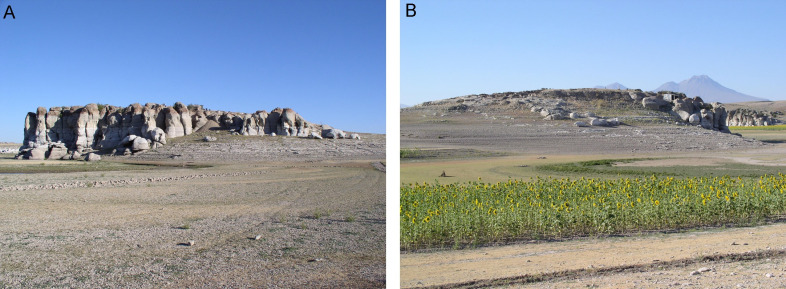
Güvercinkayası site. A: Southern part of the site on the rhyodacice/ignimbrite outcop during low water level in Mamasun reservoir. B: Northern part of the site, temporarily washed by higher water level in Mamasun reservoir, Hasan Dağı volcano in the background (3.268 m) (photos by J. Řídký 2004. GK archive).

At a certain point in the site’s history, sometime before the end of the MCH (before 4,820 – 4,750 cal BCE), the area of Güvercinkayası (hereinafter referred to as GK) was strictly divided into two areas - the one in the south, protected by the fortification wall and high rocks (Fig 2; S1 Video), is sometimes referred to as the citadel or the upper settlement while the area in front of the fortification wall is referred to as the lower settlement (Fig 1; e.g., [4,5,28]). This visually striking demarcation of one particular section of the settlement area gives rise to several interpretations. For example, it may be a material manifestation of the presence of some form of elite who sought to define their privileged space (see, e.g., [4]). However, it may also represent a well-thought-out strategy of the entire community to protect important common property (storage, or some other special purpose area). This second model has already been proposed for a similar MCH context at Yumuktepe-Mersin (Level XVI), in the south of Central Anatolia (5000 – 4600 cal BCE; e.g., [29]).

The GK site was completely excavated between 1996 and 2017 and, thanks to the well-preserved remains of segmented structures and the large number of finds, it offers a suitable opportunity for studying the activity areas and social structure of the settlement [4,5,16]. In this work, we will use MAs to test which of the aforementioned hypothetical possibilities is more likely and to what extent such artifacts are significant with regard to this issue. In the following sections, we will first classify and describe the MCH items within the MA assemblage. In several cases, using use-wear analysis, we will verify whether all the objects in defined MA groups were used for any kind of work or for other purposes. We will look at which raw materials were used for MA production, determine whether they can be locally sourced or not, and evaluate the condition of the artifacts. In the last and most important part of the work, we will try to penetrate the wall of time using statistical and spatial analysis of selected morphometric groups to answer several socio-economic questions:

• Is it possible to define any significant patterns (e.g., shapes, dimensions, preservation, quantity) in the upper settlement (citadel), protected by its fortification wall, and do these patterns differ from those in the rest of the excavated settlement, thus pointing to the presence of a person, group, or groups with a special status?
• Are classified MA groups made from various raw materials equally distributed throughout the excavated area of the settlement? Or can we localize any special activity areas with a focus on morphometric groups, and what does this tell us about the social structure?

## Materials and methods

### Summary of information about the site

The completely excavated GK site covers an area of 3,600 m². The settlement is located on a 20 m high ignimbrite/rhyodacite rock (1106.08 m above sea level) near the Melendiz River, with the terrain sloping slightly to the north and northeast (Fig 2; S1 Video; S1 Fig ). This part of the site has been destroyed by high water levels caused by the Mamasun reservoir, and it is therefore not possible today to determine the original spatial extent of the settlement. Today, the investigated part of the site can be vertically divided into several construction-chronological levels (S1 Fig). While the very first activity on the site is represented by lines of simple post holes dug into the rock, from Level I to Level II a number of stone structures and a high concentration of objects were documented. According to findings so far, Level I and Level II (both MCH, which can only be distinguished with difficulty because the stone structures are connected to each other; 5,200 – 4,820/4,750 cal BCE) represent a local building style consisting of rectangular, stone-walled structures with flat roofs. The final level, Level III (Late Chalcolithic; 4,700 – 4,400 cal BCE; according to the relative chronology based on pottery it may last until 4,200 BCE), however, is characterized by a completely new building phenomenon, considered to be a Late/Post Ubaid influence, associated with mud brick architecture and some new types of artifacts [4,30]. Unfortunately, due to surface erosion, not many intact architectural remains survive from Level III, and the dating of some artifacts to this time, including about five MA pieces, is also problematic. In contrast, the previous MCH Level II, which apparently came to an end as a result of a huge fire in the southern part of the site, offers many opportunities for studying all types of finds [5]. Unfortunately, a complex evaluation of most categories of artifacts and biofacts is currently lacking. Results so far, based on interim reports or student theses, are mostly summarized in the cited work [5]. At the moment, it is not possible to conclude whether the settlement was terminated on purpose (for example, for ritual reasons) or after some kind of disaster.

**Fig 3:**
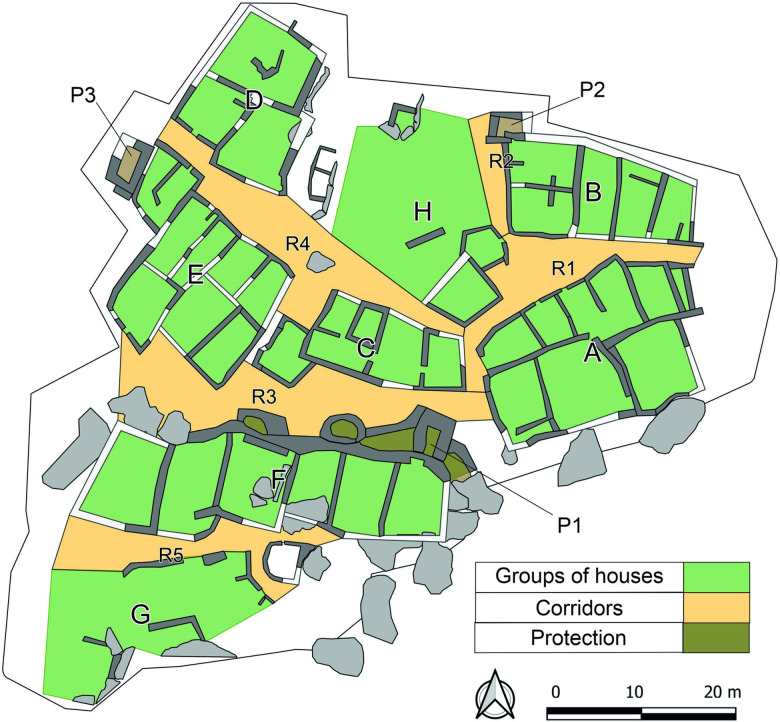
General plan of the MCH Güvercinkayası site with the units mentioned in the text. A-H: Groups of houses; R1-R5: Corridors; P1-P3: Protection walls. Map by D. Pilař.

During the roughly four hundred years of occupation of the site in the MCH, several dozen buildings/houses were constructed in the exposed part of the settlement, some of which are better preserved than others. According to the current state of knowledge, we are unable to verify if all parts of the MCH settlement functioned together at the same time. Based on the horizontal stratigraphy and general plan of the settlement, this is likely. Buildings/houses that share walls were spatially organized into eight clusters, described as groups A-H (Fig 3; [30]). Groups A-H are defined by five through corridors, labelled R1-R5. Most importantly, a massive fortification system, consisting of walls and two towers, was added to the rear (northern) walls of the houses in the southern, highest part of the site: these houses constitute group F. This entire upper settlement, formed by group F and by the badly preserved group G, separated by corridor R5, is protected on the other side by high rocks. It is likely that the lower settlement was also fortified by a wall at some point [4].

Storage spaces such as storage rooms (about one third of the interior area of the houses), storage bins and storage vessels, are an integral part of all better-preserved buildings/houses during the MCH [4,5]. According to the presence of fire features, such as ovens and open hearths, the vast majority of buildings fulfilled a residential function, so we will use the term houses. However, what is striking and important for the focus of this work is the increased amount of all types of storage structures in group F in the upper settlement [5]. Apart from the mentioned passage corridors R1-R5, separating groups of houses, no larger open communal places are recorded either in the upper or in the lower settlement. However, most of the daily activities may have taken place on the roofs, which may have been common areas to all houses within a particular group [16]. We assume that the uncovered GK findspots include places where MAs were stored (inside houses) and where they were used (inside houses and/or on their roofs) [16,31,32]. Contexts with complete grinding tools correspond to this assumption [16]. Apart from the slightly larger houses that make up group F, there are no other visually striking buildings anywhere within the entire settlement.

According to the results so far, the local economy of the MCH period was based on animal husbandry – specifically on pastoral grazing of *ovicaprines*, especially sheep [33–35]. It is assumed that the inhabitants engaged in the production of milk, meat, and wool. Milk processing was also confirmed by the identification of lipid residues on sherds across the site [36]. Based on the previous study of skeletal remains and butchery waste from different parts of the site, it seems that there is variety in the animal remains from the lower settlement, while in the upper settlement bones attesting to higher quality meat are more often found [4,37]. However, according to previous conclusions, the local economy was also based on dry, rain-fed farming. The archaeobotanical findings have not yet been comprehensively evaluated. Preliminary results from several samples suggest that wheat (*Triticum turgidum ssp. dicoccon*), barley (*Hordeum vulgare*), lentils (*Lens culinaris*), bitter vetch (*Vicia ervilia*) and grapevine (*Vitis vinifera*) were used [30].

The archaeological assemblage includes ceramic artifacts, such as diverse vessel shapes and zoomorphic and anthropomorphic figurines. Bone tools, mainly awls and needles, were also identified. Additionally, a substantial amount of obsidian (and less often flint) artifacts were recovered. These include raw materials, knapping debris, and finished tools like retouched blades, sickle blades, and scrapers. Obsidian was further utilized for crafting mirrors (five instances), beads, pendants, and the eyes of ceramic figurines [38,39].

### Geological setting

**Fig 4:**
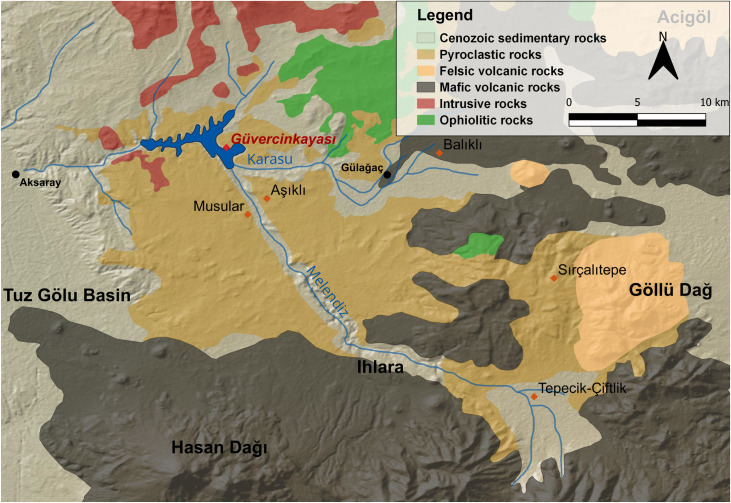
Geological map of the study area. Güvercinkayası is highlighted in red. Red points: other prehistoric sites in the vicinity; black points: modern cities. Modified from [45–47]. Map by K. Doležalová, background map: Global Multi-Resolution Topography Synthesis (GMRT; [48]).

The GK site lies in the ignimbrite field that is part of the Cappadocia Volcanic Province (CVP, also termed the Central Anatolian Volcanic Province; Fig 4). Ignimbrite is a pyroclastic rock type originating from the eruption of volatile-rich dacitic to rhyolitic magmas. In Cappadocia they formed large flow deposits, which were interbedded with pumice-fall layers [40–44]. Unlike the lower soft layers, the upper parts of the ignimbrite sequence in the CVP are characteristically strongly cemented rocks, which are resistant to weathering and feature columnar jointing [42]. Therefore, the ignimbrite field developed into an astonishing badland landscape with scenically weathered ridges and erosional chimneys. Along the course of the Melendiz River, deep dissected valleys, such as the Ihlara Valley, have been formed by elevated rock outcrops.

**Fig 5:**
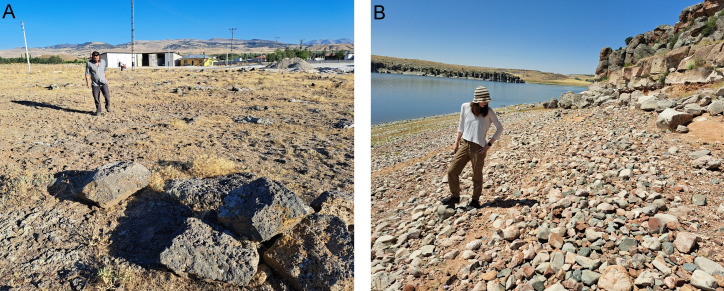
Different typical accessible raw material sources in the micro-region. A: Vesicular basalt outcrops near Gülağaç town, 10 km from the site; B: Natural pebbles and cobbles of various raw materials and sizes in the riverbank of the Melendiz River near Çatalsu village, close to the site (photos by J. Řídký 2023).

Ignimbrites erupted within the Neogene [43] and overlaid the Central Anatolian Crystalline Complex (CACC). This prevolcanic basement is characterized by Paleozoic metasediments, ophiolites, and plutonic rocks, which occur in erosional windows [43,49,50]. The ophiolites located northeast of the settlement appear in a complete sequence composed of peridotites, gabbros, basalts, radiolarites, limestones and sandstones [49,51]. These raw materials were easily accessible to the GK population, as they were found in large quantities in the form of boulders and pebbles on the banks of the Melendiz River, to where they were transported (Fig 5: B). Rocks of plutonic origin, such as granite or monzonite located to the north and west of the settlement, may have been similarly accessible on the far banks of the river. Metamorphic rocks characterized by gneisses, mica schists, amphibolites and marbles are very scarce in this area. The nearest sources are probably located at least 30 km north of the settlement near the Ağaçören Granitoid extending along the Tuz Gölü Basin [49,52].

Hasan Dağı Volcano, with its two peaks, dominates the view from the settlement to the south (Fig 2: B). The last eruption was around 7,000 BCE, and was probably witnessed by humans living in this area [45,53]. Hasan Dağı Volcano belongs to a series of stratovolcanoes in CVP, which are dominated by andesitic and dacitic lithologies [40,54]. The only two rhyolitic complexes are Acigöl and Göllü Dağ, which are responsible for the formation of lava domes and the large mass of pyroclastic deposits [45]. On the slopes of Göllü Dağ are outcrops containing huge masses of obsidian, which was traded over long distances [55].

In the area along the Melendiz River and its tributaries, the rocks are interbedded with fluvial and alluvial sediments. The Melendiz River flows to the Tuz Gölü Basin, where it forms a large alluvial flood plain. The Tuz Gölü Basin, partially filled by the lake, is separated from the Cappadocian plateau by the footwall block of the mountain front characterized by a series of alluvial fans [46]. The sedimentary sequence of the Tuz Gölü Basin consists of carbonate rocks, evaporites, and terrestrial clastics [56]. This area is considered to be a very important source of salt [57].

### Macrolithic artifacts

MAs are tools or other objects, mostly (but not exclusively) with ground surfaces, which are made from specific raw materials valued for their hardness, tenacity, durability, texture, and perhaps also for their color (e.g., [3,11,20,21,58–61]). A significant proportion of these artifacts were used as tools for cracking, pestling, grinding-milling, pounding, polishing, but also for abrading and the reduction of materials, and for other activities, such as the preparation of plaster, for example [62]. Some of the tools were used in pairs, as bipartite devices (lower-upper stone sets, mortar-pestle sets; e.g., [18]), but others, much simpler, could represent *ad hoc* selection for their suitable shape, size and raw material for multiple activities (i.e., hammer stones, pounding stones, polishing stones and a combination of these). Some MAs weigh several tens of kilograms, and if there were no sufficiently suitable raw materials with the required properties for their production in the immediate vicinity of the settlements, their provision and transport would have required a certain logistical effort and expenditure of energy. They may therefore have been highly valued objects, occurring in specific parts of the settlement (e.g., [24]). Besides their role in adaptation strategies, subsistence strategies, eating habits and cooking practices, or various craft productions, MAs are in general very important indicators of settlement structure, socio-economic relations, social stratification, or ritual behaviors of past societies (see, e.g., [11,22,24,26,63–67]).

Different analytical approaches are used for MA classification, such as morphometric analysis, raw material determination, use-wear analysis combined with experimental programs, analysis of microresidues (phytoliths, starch grains), and contextual studies (e.g., [3,6,18,21,64,68,69]). In this work, we focus on morphometry, macroscopic raw material determination, in several cases on use-wear traces, and on quantitative trends and contextual variables of MAs (see below).

### Dataset

The assemblage we had at our disposal totaled 1793 pieces (weighing a total 5404.3 kg; S1 Dataset). However, it was originally estimated that there would be about 100 more recorded MAs. A total of 68 pieces could not be assigned to any research season and no contextual information was available for them. Some of the artifacts are stored in Aksaray museum, where they are part of the permanent exhibition. We had the opportunity to see this group of artifacts in 2023, and apart from two completely preserved axes, on display in the museum, they do not differ fundamentally in terms of shapes or raw materials from the morphotypes we studied. For approximately five MAs, which could theoretically belong to Level III of LCH, their secondary use from earlier MCH layers cannot be ruled out.

### Classification

**Fig 6:**
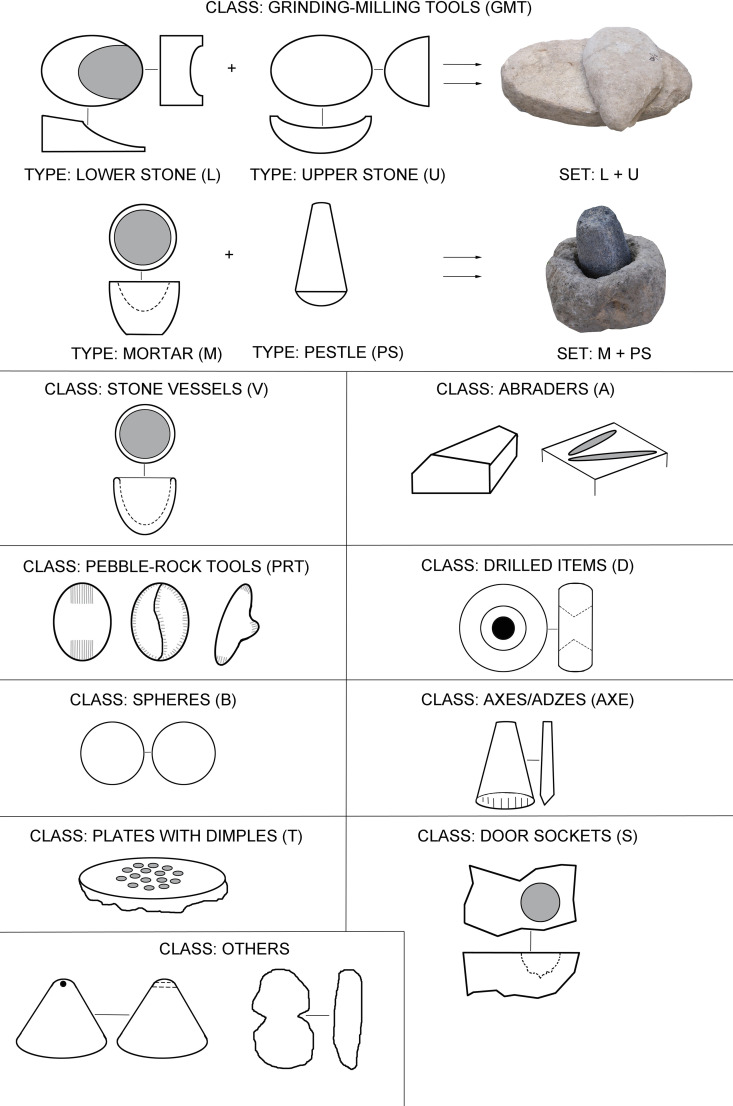
Major classes, types and sets of macrolithic artifacts (MAs) in Neolithic and Chalcolithic assemblages from Central Anatolia. See also Table 1. Modified after J. Řídký [70].

**Fig 7:**
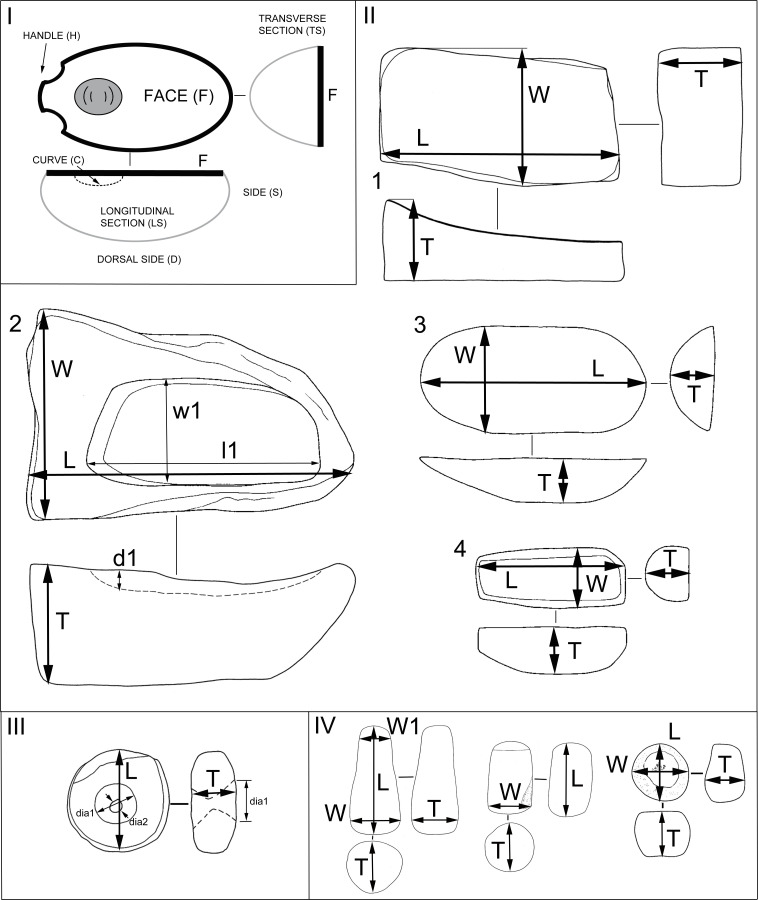
Metrics. **Terminology (I) and metric variables (II-IV) used.** I: Terms used for description; II. Metric system for GMTs (1–2: lower stones, 3–4: upper stones); III. Metric system for drilled items; IV. Metric system for pestles and pebble and rock tools. L – max. length; W – max. width; T – max. thickness; d1 – max. depth of the basin; l1 – max. length of the basin (for lower stones and mortars). Modified by J. Řídký after [3].

Artifacts were classified with respect to shapes, to basic dimensions, with a focus on the method of production and based on the location and shape of the utilization areas (Fig 6; S1 Text). The method of collecting metric data and the basic terminology were adopted and modified from the work of K. Wright, J. Adams, and other experts (Fig 7; [2,3,70]).

**Table 1:** List of macrolithic artifacts (MAs).

Abbreviation	Description	Total	%	Ʃ Complete p.	% Complete p.
A	Abrader	43	2.40	39	3.43
AXE	Axes and adzes	6	0.33	2	0.18
B	Stone sphere	136	7.59	130	11.43
D	Drilled item	48	2.68	21	1.85
L	Lower stone	391	21.81	116	10.20
LM	Lower stone combined with mortar	5	0.28	2	0.18
M	Mortar	85	4.74	47	4.13
OTHER	Unclassifiable in form (flake, raw material supply, pigments, etc.)	33	1.84	20	1.76
PRT	Pebble and eroded rock tools (multiple wear traces)	488	27.22	419	36.85
PS	Pestle	10	0.56	10	0.88
R	Roughout - cannot be determined more precisely	2	0.11	0	0.00
RD	Roughout of drilled item	18	1.00	9	0.79
RL	Roughout of lower stone	7	0.39	5	0.44
RM	Roughout of mortar	3	0.17	2	0.18
RU	Roughout of upper stone	29	1.62	22	1.93
S	Door socket	29	1.62	24	2.11
T	Plate with dimples	10	0.56	6	0.53
U	Upper stone	431	24.04	259	22.78
UL	Upper stone combined with lower stone	4	0.22	2	0.18
V	Stone vessel	15	0.84	2	0.18
**∑**		**1793**	**100.00**	**1137**	**100.00**

Abbreviations used in the text (in alphabetic order) and numbers and percentages of individual finds. Complete p. means complete pieces. Authors J. Řídký, D. Pilař, and K. Doležalová.

For individual items, we noted whether they were complete or incomplete (the occurrence of striking points on fragments was also noted), the estimated percentage of preservation, basic dimensions (maximum length, width, thickness; rounded to centimeters) and weight in kilograms (or grams for some smaller items). Furthermore, the raw material was macroscopically determined. A systematic comparative analysis of the stone raw materials of artifacts and surrounding outcrops and other sources of materials has not yet been carried out. Raw materials used for MA production were determined macroscopically, based on standard geological classification (granularity, texture, structure, cohesion, porosity, and minerals) as well as by measuring magnetic susceptibility. Some of the raw materials were described in one of the previous works [16]. Artifacts were divided into individual classes and, when possible, into types and subtypes, either on the basis of shape characteristics, or according to the occurrence of typical macroscopically determinable traces of use. This division at the level of subtypes is the most important one for spatial analyses (see below).

The artifacts in our assemblage were classified into the following basic groups (see Figs 6-7, Table 1, and for detailed classification system S1 Text):

#### Class: Grinding-milling sets (GMT).

These are sets of two compatible types of tools, which can be divided either into lower stone-upper stone sets or into mortar-pestle sets.

**Fig 8:**
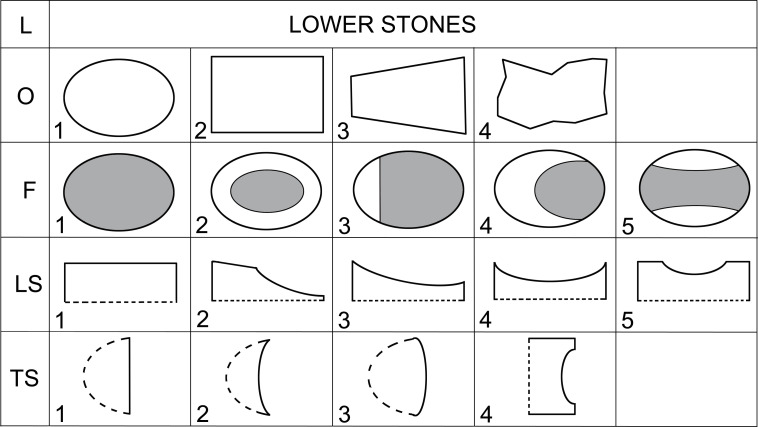
Description system for L-types (lower stones) of GMT-class. Description of the outline of the artifact in plan (O), description of the position of the face (F) and outlines of longitudinal (LS) and transverse (TS) sections. Modified after J. Řídký [70].

**Fig 9:**
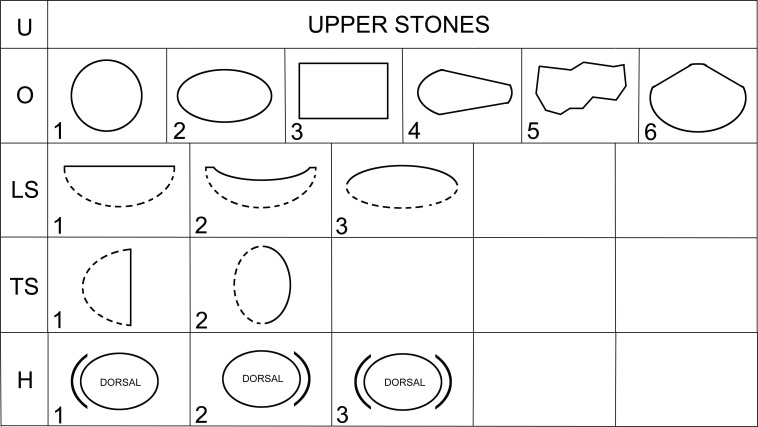
Description system for U-types (upper stones) of GMT-class. Description of the outline of the artifact in plan (O) and outlines of longitudinal (LS) and transverse (TS) sections. Position of handles (H) on the left, right or on both sides from a view to the dorsal side. Modified after J. Řídký [70].

Lower stone types (L) are passive tools of lower-upper stone sets (L + U), usually reaching about 50 cm in length and 30 cm in width (Fig 8). In some cases, it is possible to distinguish a lower stone and mortar that were used in combination (LM), or a lower stone and upper stone that were used in combination (UL). Upper stone types (U) usually measure up to 30 cm in length and 20 cm in width and are an active tool of the lower-upper stone sets (Fig 9).

**Fig 10:**
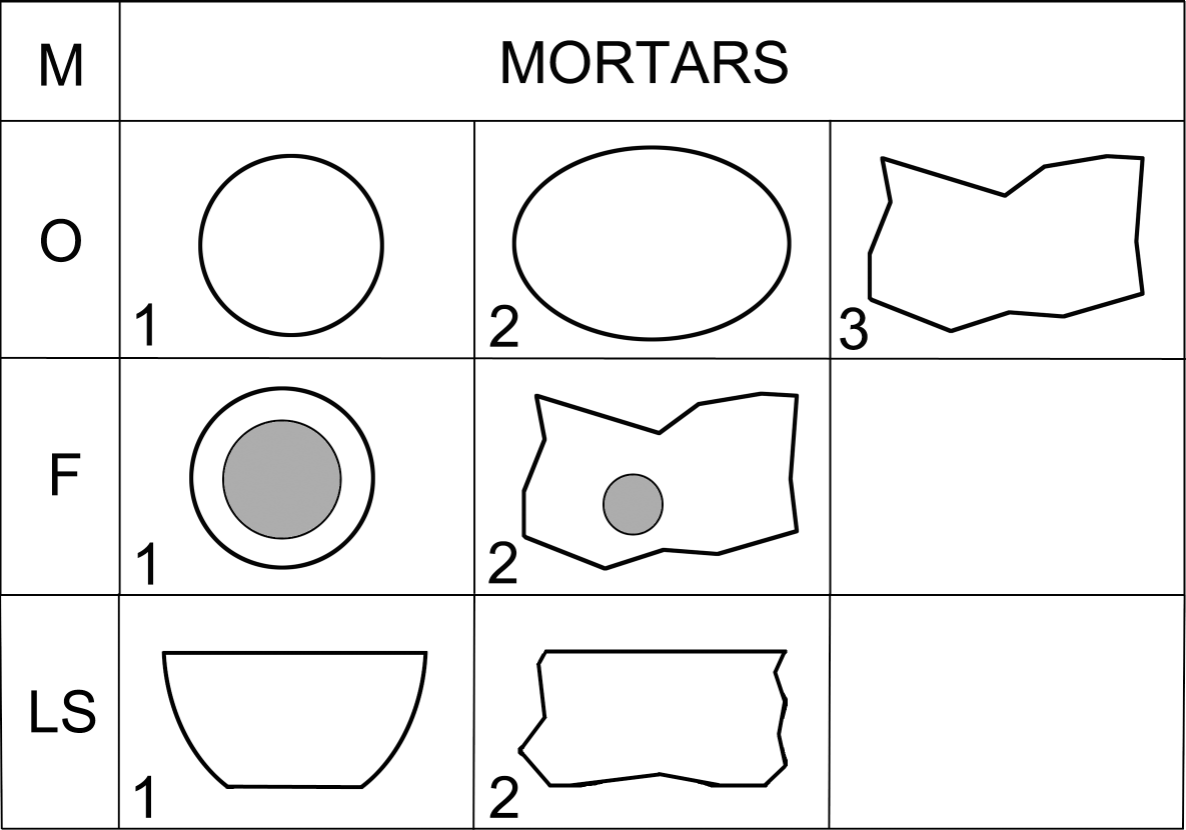
Description system for M-types (mortars) of GMT-class. Description of the outline of the artifact in plan (O), description of the position of the face (F) and outline of longitudinal (LS) section. Author J. Řídký.

**Fig 11:**
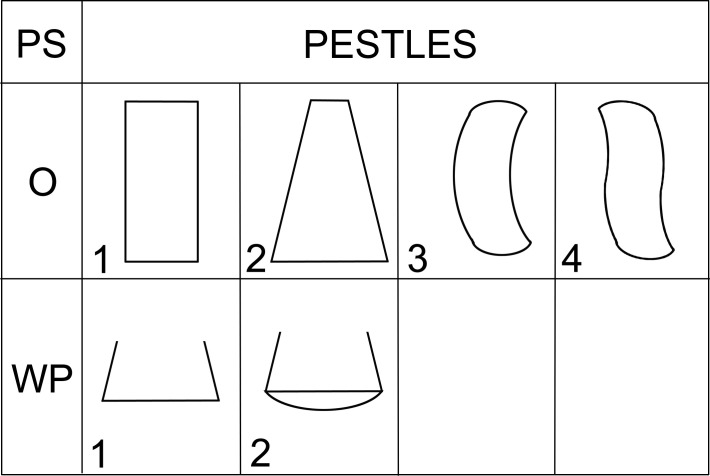
Description system for PS-types (pestles) of GMT-class. Description of the outline of the artifact in plan (O), description of the outline of the working part (WP) in plan view. Author J. Řídký.

Mortar types (M) are passive tools with massive walls, part of mortar-pestle sets (M + PS), measuring up to about 30 cm in length and 30 cm in width, with a circular face (Fig 10). The face is always regular and concave in transverse section. Pestles (PS) are active tools, compatible with mortars. PS-types in the GK assemblage are oblong or irregular in shape (Fig 11), with an oval cross-section and reach a length of about 20 cm and a width of up to 10 cm.

#### Class: Vessels (V).

Items in this class have a maximum length of about 20 cm and a width of 15-20 cm. They include deep bowl-shaped vessels with thin walls (compared to mortars), with an oval or circular face. In the vast majority of cases they are made from softer raw materials although there are some exceptions to this rule. The surface of the V-class is carefully treated by fine grinding or polishing.

#### Class: Abraders (A).

**Fig 12:**
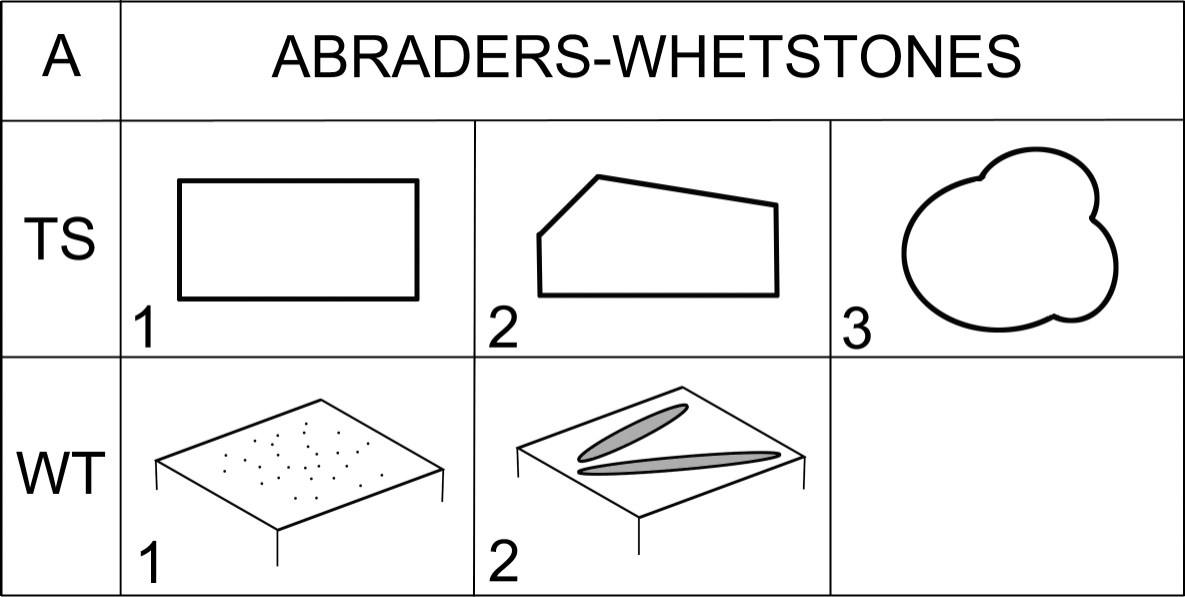
Description system for A-class (abraders). Description of the outline of the artifact in transverse section (TS), and according to use traces (WT), Author J. Řídký.

This class includes items that bear various abraded surfaces, or very typical straight U-shaped grooves (Fig 12), and they are usually made of materials suitable for reducing various materials. Abraders in the GK assemblage usually reach a length of about 30 cm and a width of about 20 cm.

#### Class: Multiple-use tools (PRT).

**Fig 13:**
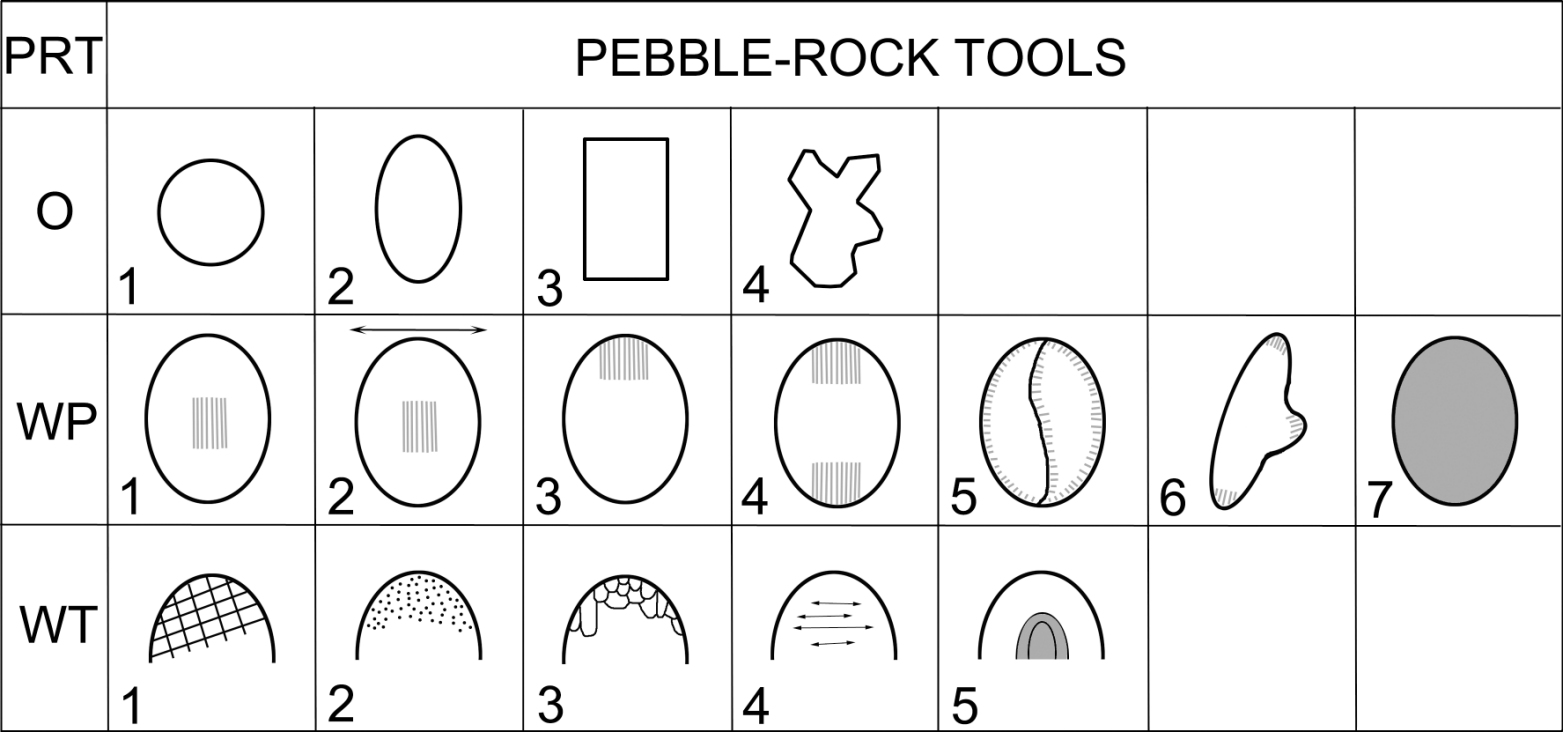
Description system for PRT-class (multiple-use tools). Description of the outline of the artifact in plan (O), description of the position of the working part (WP) in plan view, and according to type of use-wear (WT). Author J. Řídký.

This quantitatively rich and morphologically broad class of simple artifacts (Fig 13), with a maximum length of about 15 cm and a width of about 10 cm, usually did not require any complex shaping (pebbles, cobbles, suitable pieces of eroded rocks). In our classification system, we do not divide this class into types in the first step of analyses (polishers, percussion tools, etc. [6–11]), because most often these are multifunctional tools bearing multiple traces of use, in different utilization areas.

#### Class: Drilled items (D).

**Fig 14:**
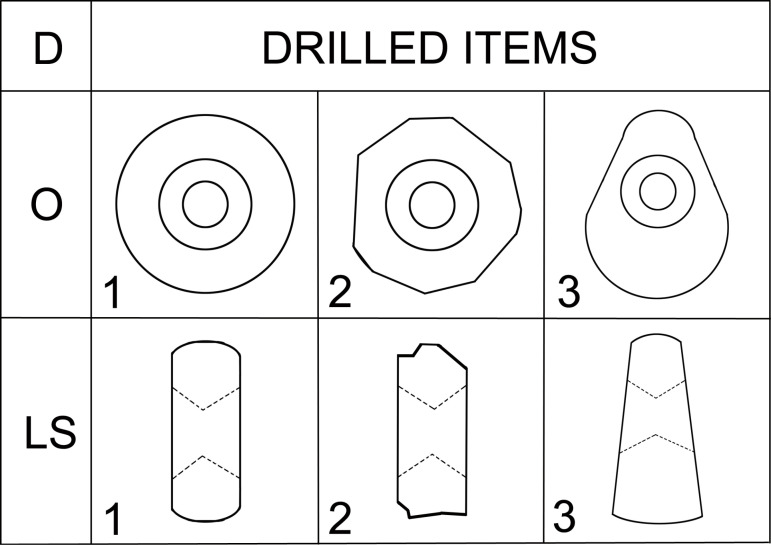
Description system for D-class (drilled items). Description of the outline of the artifact in plan (O), description of the outline of the longitudinal section (LS). Author J. Řídký.

The artifacts that constitute this class may be round, oval or sometimes irregular in shape (Fig 14) and have a maximum length of 15 - 20 cm and a width of 15 - 20 cm; their essential distinguishing is a perforation, probably produced by drilling.

#### Class: Stone spheres (B).

This is a very specific class made up of almost regular ground stone spheres, which have a maximum diameter of 2 cm to 10 cm.

#### Class: Plates with dimples (T).

These are variously shaped plate formed pieces of rock, mostly of oval shape, less often of irregular shape, bearing a number of spatially organized dimples.

#### Class: Door sockets (S).

These are variable in shape and are mostly made from local raw materials. The occurrence of holes with different diameters and irregularly shaped transverse-sections is significant. The bottom of the irregularly concave active surface is usually flat.

#### Class: Axes/adzes (AXE).

This class of tools, originally with cutting edges, is rare in the GK assemblage. We recorded only two pieces with a preserved cutting edge. They were mostly secondarily used as hammer stones.

#### Class: Others.

A group of rare finds. For example, variously shaped stones of local ignimbrite, apparently serving as binding stones, are included here.

Some classes (Table 1) are presented here only to provide a general idea of the morphological trends in MCH. For example, portable S-class artifacts were part of the architecture, and therefore we will not consider them in this work except to indicate the numbers present. Also, tools with cutting edges, such as axes/adzes, form a small group within the collection, and moreover, except for the two pieces stored in Aksaray museum, they were mostly used secondarily as hammer stones (in our database, hammer stones are part of the PRT-class).

### Microscopic use-wear analysis

In what follows, use-wear analysis will serve to supplement basic information on the possible functional use of several individual groups (see below); much deeper analyses are needed with larger amounts of different subtypes, raw materials, and reference collections. The types of substances that were ground have been somewhat neglected due to the fact that our own experimental reference collection has not yet been published. The focus is on distinguishing which shapes of artifacts were used (if at all) and in what way.

**Fig 15:**
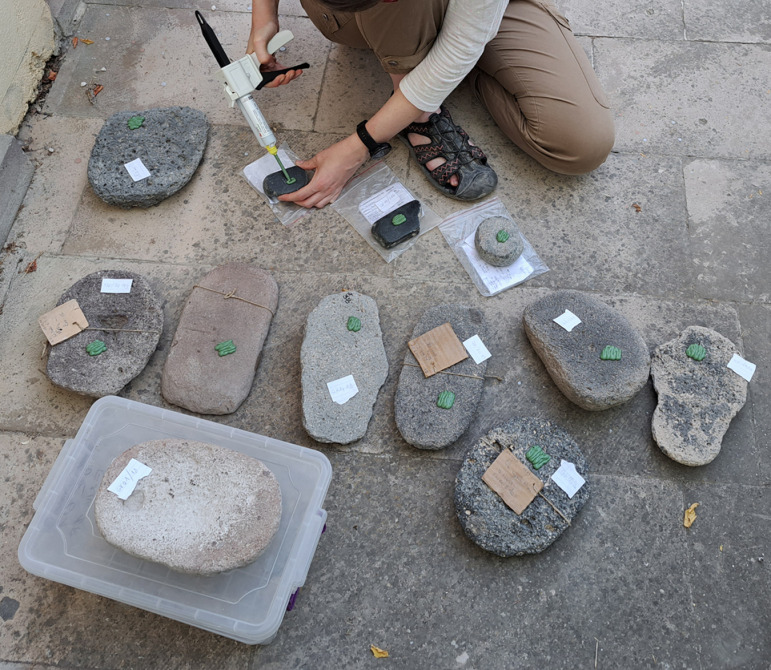
Application of high resolution silicon casts on the utilization surfaces of MAs, mostly of U-types of GMT-class. Photo by J. Řídký 2023.

The shape of MAs is influenced by many circumstances, such as the availability and characteristics of raw materials, human skills, traditions, and cross-community interactions. Multiple shapes can lend themselves to the same functional use and the same shape can have many functional applications [21,71]. Therefore, twenty MAs were chosen for use-wear analysis to better understand the kinematics and life histories of the tools, to explore how function is connected to the shape or raw material and to determine whether the tools were used at all. A reference collection of experimental tools made from local raw materials is not yet available for comparative analysis of use traces. However, the study is based on published reference collections[20,72–76] and our experimental program conducted on rhyolite GMTs [69]. The artifacts for the use-wear analysis were selected according to the state of preservation (only unburned and complete pieces) and the objectives of the research questions. At least one completely preserved sample was selected from each class. The main aim was to have an equal number of comparable artifacts from the upper and lower parts of the settlement. A varied raw material representation with published experimental reference collections was preferred [2,69,77–79].The artifacts were first analyzed macroscopically in Türkiye using external low-angled light, which was combined with the microscopic observation at low levels of magnification using a DINOLITE digital microscope. Subsequently, high resolution silicon casts were taken of the tools’ utilization surfaces (Fig 15) (3M™ Express™ Light Body Regular Set VPS Impression Material). These casts were then transported to the Czech Republic and investigated under an Olympus BXFM Optical Microscope. The terminology for use-wear description was taken from previously published papers [2,69,77–79].

### Spatial analysis

After considering all the information presented in the summary of information about the site, and taking into account some earlier attempts at contextual and spatial analysis [16,30,80], we worked with spatial analyses in our study under the assumption that:

i) The vast majority of the collected MA assemblage comes from Level II, i.e., from the very end of the settlement in the MCH period, which coincided with a huge fire in part of the settlement;
ii) the objects were found approximately in the places, or in the immediate vicinity of the places, where they were stored or even used;
iii) that even though they are objects from different depths of the fills of individual structures, they still come from approximately the same time. MAs from the upper layers of some structures may belong to the collapsed roof contexts [16] and therefore belong to the relevant group of houses.

To answer our goals, we applied spatial analyses at the level of:

• **Upper settlement** and **lower settlement**, strictly separated by a fortification wall (Fig 1).
• In the next part of the work, we searched for significant patterns at the level of these **units** (Fig 3): Groups of houses (A-G), corridors (R1-R5), and other spatially separated smaller structures, in this case P1 (space inside of the whole fortification system) and P3 (another possible fortification structure on the north-west edge of the settlement).

During the analyses, the isolated units were compared on two levels. The relative frequency of individual types and subtypes was examined in order to find potential patterns. At the same time, the metric properties of types and subtypes in individual groups were compared.

For metric properties, mean values were compared using independent sample t-test (in the case of comparing two groups - e.g., lower/upper settlement) and ANOVA (in the case of more than two groups) [81–83]. In ANOVA, Tukey’s post hoc comparison method was used [82]. To perform the tests, it was first necessary to verify the normality of the data using Kolmogorov-Smirnov and Shapiro-Wilk normality tests. Another condition for performing the comparison is data homogeneity, which was determined using the Levene’s test [82]. However, the datasets examined often did not meet the above conditions. The most common reasons were low numbers of artifacts in the groups, high variability of the data or inappropriate distribution of the data. Thus, in some cases, analyses have been performed on larger data sets without the fulfilment of some of the conditions, from which robust results can still be obtained [82,84].

The representation of the artifacts in each of the groups was observed empirically after being graphically displayed in a GIS. The groups were then analyzed using factor analysis to find patterns in artifact composition [85,86]. Standardized data were used for factor analysis [87] and the varimax method was used for factor rotation [81,86]. Finally, clusters of groups that had similar factor loadings in the factor analysis were displayed on a map using GIS.

Spatial analyses were performed using MS Excel, Jamovi and SPSS software.

## Results

### Raw materials of the GK assemblage

The selection of the raw material used for various MAs depended on many factors. However, given the difficulties of transport, distance from the source was one of the important criteria [6,24]. Local sources include raw materials collected at a distance of up to 5 km. Regional sources are located at a distance of 6 to 20 km and supra-regional sources must have been transported from more than 20 km away [88].

**Fig 16:**
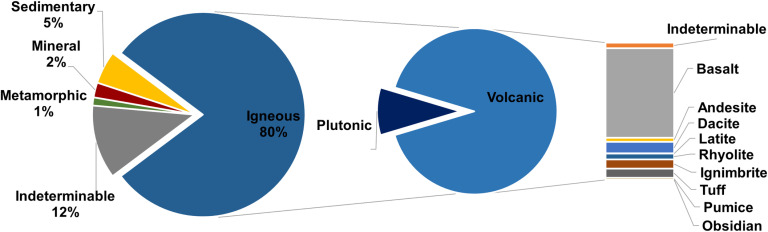
Overview of raw materials used for MA manufacture (n =  1793). Author K. Doležalová.

The raw materials used for MAs at the GK site were predominantly of volcanic origin (Fig 16; S1 Table), which is to be expected given the location of the site in a volcanic region. Sedimentary and metamorphic rocks were seldom used as raw materials. The diversity of volcanic rock exploited is large, although basalt clearly dominates. In total, more than 3.5 metric tons of basalt were found at the site and had to be transported to the settlement. Basalt occurs mainly as a variant with vesicular texture. This hard cohesive rock with sharp-edged pores was considered the most suitable for making grinding stones in many parts of the world [89–92]. Likewise at GK, vesicular basalt was the most used raw material for the production of GMTs (Fig 16). The nearest sources of vesicular basalt are probably located near the present day village of Gülağaç, approximately 10 km from the settlement as the crow flies (Fig 4; Fig 5: A). Other volcanic rocks, namely dacite, rhyolite and andesite, were also used, but the locations of the exploited outcrops are uncertain. Local raw materials, such as ignimbrite, tuff and pumice were rarely used because of their relative softness. Only ignimbrite mortars have been found more often. On the other hand, pestles, which are not common at the settlement, were made primarily of vesicular basalt. The other variety of basalt with compact structure occurs in much smaller quantities and it was predominantly used for smaller artifacts, such as PRT-class. Sources of compact basalt could be found either to the northeast in the ophiolitic sequence or to the east as a part of the basalt lava flows. However, it also occurs in large quantities on the banks of the Melendiz River. Other raw materials such as gabbro, diorite and quartz are also found on the banks of the Melendiz. Occasionally used sedimentary rocks, such as limestone, sandstone and conglomerate, had to be procured probably within a range of 20 km to the northeast where they form part of the ophiolite sequences, or to the west in the Tuz Gölü Basin. It seems that only metamorphic raw materials were sourced over very long distances. However, the occurrence of metamorphic rocks in the assemblage is very limited and insignificant. The only exception is marble vessels (V-class), where the raw material had to be purposefully sourced and imported. The nearest sources are probably to the north, more than 20 km from the settlement.

In summary, most MA groups (PRT-class, A-class, B-class, T-class, and partly also M-type of GMT-class) were predominantly made of local raw materials, which encompass pebbles/cobbles gathered from the riverbank (gabbro, compact basalt, quartz) and rocks from nearby volcanic outcrops (ignimbrite, tuff and pumice). However, the largest amounts of raw materials come from regional sources. These are mainly vesicular basalt and sedimentary rocks used for GMT manufacture. The only significant raw material representing a probable supra-regional source was marble.

### Morphometric groups of macrolithic artifacts

More detailed information on metrics can be found in the tables for more numerous classes or types. For groups with lower numbers of objects, we mention the lowest and highest metric values.

### Grinding-milling tools

Among the raw materials used to manufacture GMT class artifacts, volcanic rocks clearly predominate, of which vesicular basalt has the highest representation. Out of a total of 371 of the L-types determined, vesicular basalt represents 67.39% (n =  250) of all raw materials. Next in frequency is dacite, which accounts for almost 10% of raw materials used. Vesicular basalt predominates in the raw material of U-types, representing 60.23% (n =  156) of the 259 pieces determined. The representation of other raw materials is a little different than in the case of L-types. Among the raw materials used, dacite, compact basalt, but also sandstone (ophiolite), rhyolite, and andesite occur more often. Of the 82 pieces of M-types whose raw material is determined, vesicular basalt makes up less than half (47.56%; n =  39). Local ignimbrite (25.61%; n =  21) comes second among raw materials used for M-types. Various raw materials, in boulder/pebble form, were used for PS-types; basalt is the most common raw material in this numerically small group of tools.

According to macroscopic observation, the majority (c. 75%) of L-types in the GK assemblage were manufactured by flaking (n =  305; 76.1%). Complete L-types tend to vary between 28 cm and 44 cm in length, between 20 cm and 31 cm in width, between 7 cm and 14 cm in thickness, and between 6 kg and 19 kg in weight (Table 2). However, L-types also include small pieces, with a length of only 10 cm, as well as the largest of all MA pieces found at the site, reaching a length of up to 65 cm and a weight of 75 kg.

**Table 2:** Comparison of length, width, thickness (all in cm) and weight (in kg) of the L-types of GMT-class.

Cases	Min.	Max.	Mean	Std.Error	Median	Std.Deviation	QI	QIII	IQR	Range
**LENGTH**										
118	10	65	36,67	1,043	37	11,335	27,75	44,25	17	55
**WIDTH**										
118	8	60	25,82	0,713	24	7,746	20	31	11	52
**THICKNESS**										
118	1	26	11,14	0,476	11	5,174	7	14	7	25
**WEIGHT**										
118	0,33	75	14,92	1,294	11	14,057	5,96	19,13	13,2	74,67

QI =  lower quartile (25%); QIII (upper quartile (75%); IQR =  interquartile range. Author J. Řídký.

The majority of U-types were manufactured by fine flaking, and in total only 5% (n =  23) were ground. The length of complete U-types oscillates most often between 21 cm and 26 cm, width between 14 cm and 18 cm, thickness between 3 cm and 15 cm, and weight between 2 kg and roughly 3.5 kg (Table 3). Only seven pieces are smaller than 15 cm, which is roughly the limit at which they can be comfortably held in one hand, or in two hands placed above each other, during operations. All the other completely preserved pieces were evidently held with both hands side by side during use.

**Table 3:** Comparison of length, width, thickness (all in cm) and weight (in kg) of the U-types of GMT-class.

Cases	Min.	Max.	Mean	Std.Error	Median	Std.Deviation	QI	QIII	IQR	Range
**LENGTH**										
260	10	35	23,4	0,248	23	4,003	21	26	5	25
**WIDTH**										
260	7	25	16,14	0,162	16	2,611	14	18	4	18
**THICKNESS**										
260	3	15	6,09	0,104	6	1,675	5	7	2	12
**WEIGHT**										
260	0,44	8,5	2,9	0,087	2,6	1,408	2	3,48	1,48	8,06

QI =  lower quartile (25%); QIII (upper quartile (75%); IQR =  interquartile range. Author J. Řídký.

Among M-types, coarse flaking was identified in four cases and grinding of the body in 11 cases, the majority of the tools being finely flaked. The length of complete M-types varies most often between 18 cm and 37 cm (Table 4), width is generally between 16 cm and 31 cm, thickness between 8 cm and 16 cm, and weight between 2 kg and 20 kg. Among the complete tools, however, there are both very small pieces with a length of 9 cm, as well as large pieces, reaching a length of up to 54 cm and a weight of 56 kg.

**Table 4:** Comparison of length, width, thickness (all in cm) and weight (in kg) of the M-types of GMT-class.

Cases	Min.	Max.	Mean	Std.Error	Median	Std.Deviation	QI	QIII	IQR	Range
**LENGTH**										
47	9	54	28,26	1,763	28	12,089	18	37	19	45
**WIDTH**										
47	9	51	23,55	1,379	24	9,452	16	31	15	42
**THICKNESS**										
47	4	25	12,55	0,731	14	5,012	8	16	8	21
**WEIGHT**										
47	0,15	56	13,09	1,798	11	12,325	2	20	18	55,85

QI =  lower quartile (25%); QIII (upper quartile (75%); IQR =  interquartile range. Author J. Řídký.

Apart from one exception, PS-types are made from elongated boulder/pebble forms of various raw materials, which rather resemble pestles (perhaps they should in fact be included in the PRT-class). However, 50% of the group were ground. Their maximum length varies between 8 cm and 22 cm, width between 4 cm and 10 cm, and thickness between 4 cm and 8 cm. The lightest piece, made of andesite, weighed 0.16 kg, the heaviest one, made of compact basalt, weighed 2.3 kg.

### Roughouts of grinding-milling tools

**Fig 17:**
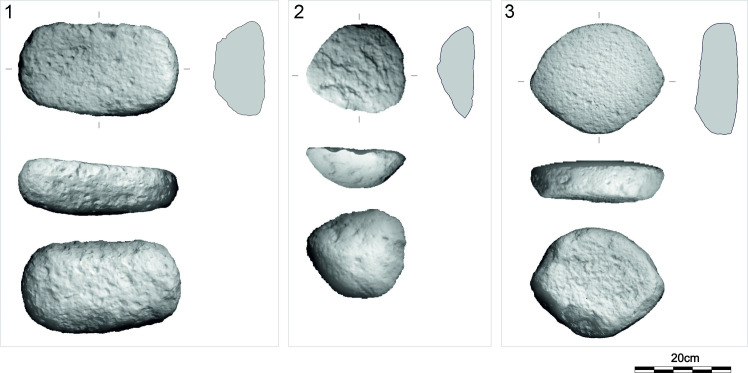
Examples of the U-type roughouts from different contexts. 1: GK10_218 (vesicular basalt; lower settlement, R4); 2: GK11_256 (andesite; lower settlement); 3: GK03_82 (vesicular basalt; lower settlement, group A). The abbreviation GK is the designation of the site, the following two digits indicate the year of the excavation, the last two digits are the inventory number of the find. For 3D models see S2 Fig. Author D. Pilař.

Roughouts of L-types (RL), U-types (RU), and M-types (RM) are present in the GK assemblage (Table 1). RU are among the most common (n =  29) roughouts (Fig 17). Although vesicular basalt predominates (n =  17; 58.62%), other raw materials also occur, such as dacite (n =  3), andesite, granite, ignimbrite, or latite. Complete RU pieces (n =  22) have a length of 12 cm to 34 cm, a width of 9 cm to 24 cm, a thickness of 3 cm to 16 cm and a weight of 1.1 kg to 11 kg. Comparing the thickness of the aforementioned used tools and their incomplete roughouts indicates there was no significant loss of mass.

RLs are mostly made of vesicular basalt and complete pieces (n =  5) reach a length of 28 cm to 58 cm, a width of 20 cm to 48 cm, a thickness of 7 cm to 12 cm and a weight of 6.5 kg to 40 kg. The similar thickness of RLs and complete L-types indicates that there was no significant loss of tool mass during use.

Only two RMs were made of dacite and local ignimbrite. These are round shapes with traces of the initial production of an active surface in the form of a round, shallow concave area. The two pieces, both of them complete, measure 34 x 30 x 16 cm (weight 12 kg) and 42 x 33 x 7 cm (weight 10 kg), respectively.

### Possible subtypes of grinding-milling tools

With the exception of PS-types, which represent only a small portion of GMT class, L-types, U-types, and also the M-types can be further divided into subtypes on the basis of shape characteristics.

### Possible L-subtypes (compare Fig 8)

**Fig 18:**
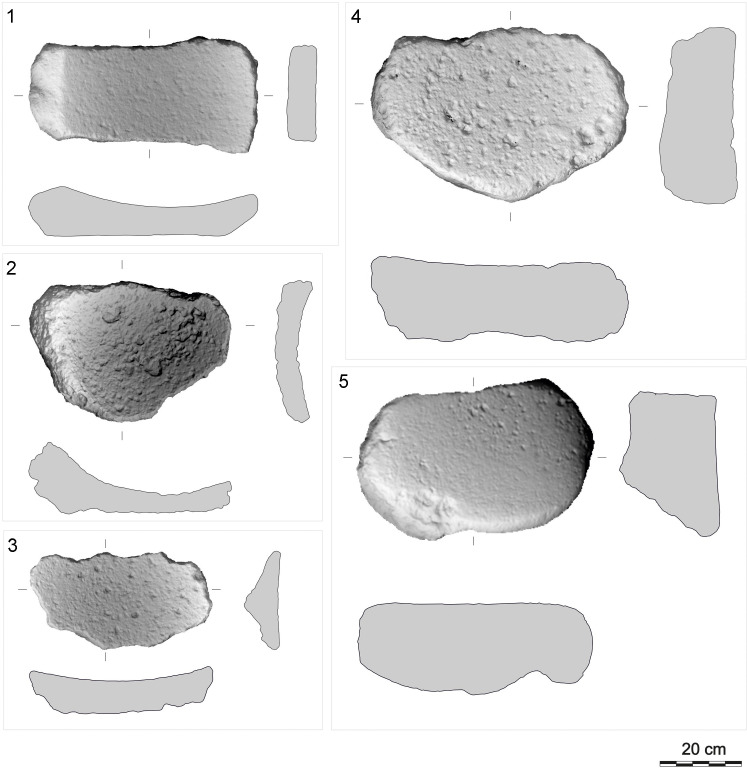
L-subtypes of GMT-class from different contexts. 1: L1-subtype (vesicular basalt; GK07_131; lower settlement, R3); 2: L2-subtype (vesicular basalt; GK09_131; lower settlement, R3); 3: L2-subtype (vesicular basalt; GK11_38; lower settlement, R3); 4: L2-subtype (vesicular basalt; GK11_329; lower settlement, R4); 5: L2-subtype (vesicular basalt; GK11_41; lower settlement, group E). The abbreviation GK is the designation of the site, the following two digits indicate the year of the excavation, the last digits are the inventory number of the find. For 3D models see S2 Fig. Author D. Pilař.

**L**1**** (n =  70) – are artifacts of all known shapes O1-O4 (Fig 18: 1, and Fig 22: A and Fig 23: E), whose active surface is spread over the entire surface of the working part (F1). LSs tend to be variously curved, except for LS5s, while TSs are always straight (TS1). Altogether 60.87% are made of vesicular basalt. The smallest complete piece measures 21 x 19 x 7 cm, the largest 51 x 32 x 24 cm. Weight varies between 3 kg and 60 kg (Median =  13 kg).

**Fig 19:**
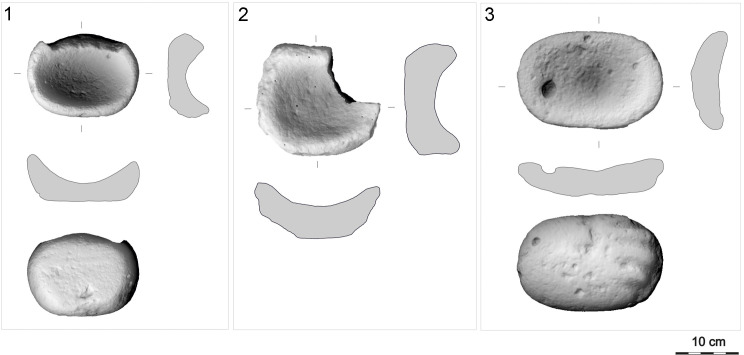
L-subtypes of GMT-class from different contexts. 1: L3-subtype (vesicular basalt; GK02_70; upper settlement); 2: L3-subtype (vesicular basalt; GK11_258; lower settlement, group E); 3: L4-subtype with modelled “hand” on dorsal side (conglomerate; GK01_18; upper settlement, group F). The abbreviation GK is the designation of the site, the following two digits indicate the year of the excavation, the last digits are the inventory number of the find. For 3D models see S2 Fig. Author D. Pilař.

**Fig 20:**
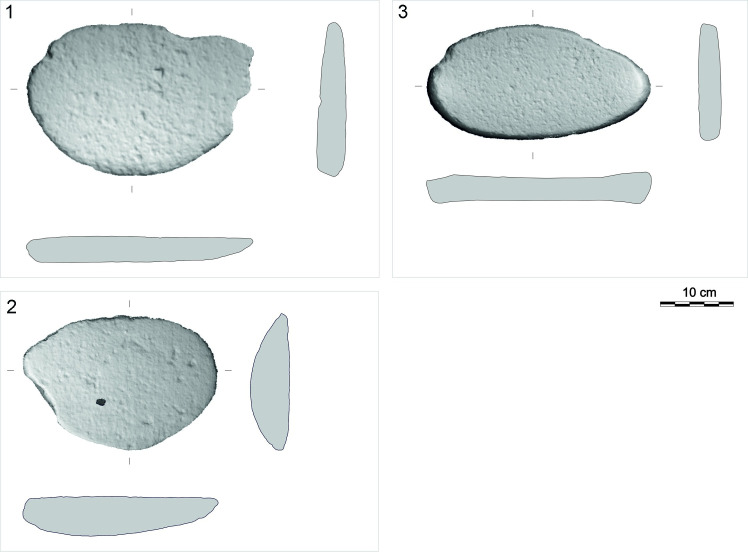
U-subtypes of GMT-class from different contexts. 1: U3-subtype with two utilization areas (vesicular basalt; GK10_71; upper settlement, group F); 2: U3-subtype (vesicular basalt; GK03_44; lower settlement, R1); 3: U4-subtype with two utilization areas (vesicular basalt; GK11_190; lower settlement, group E). The abbreviation GK is the designation of the site, the following two digits indicate the year of the excavation, the last digits are the inventory number of the find. For 3D models see S2 Fig. Author D. Pilař.

**L2** (n =  103) – is similar to the previous subtype (Fig 18: 2-5). Again the active surface covers most of the entire surface of the working part (F1); occasionally an unworn edge remains in the proximal part (F4). LSs are of various shapes, except for LS5s, but TSs are typically concave (TS2) compared to the previous subtype. Altogether 71.84% are made of vesicular basalt. The smallest complete piece measures 19 x 16 x 5 cm, the largest 65 x 31 x 23 cm. Weight varies between 2 kg and 75 kg (Median =  13.75 kg).

**L3** (n =  15) – these are invariably oval (O1; Figs 19: 1-2) or rectangular in shape (O2); other shapes do not occur. The active surface is always at a certain distance from the edges (F2), the LS is always deeply concave (LS5) and the same is TS (TS4). Compared to the previous subtypes, they are mostly finely made, of regular shape, and are smaller in size. While vesicular basalt is the predominant raw material used in the previous subtypes, in this subtype it was used for only one third of all specimens. Another difference is that only two complete pieces have survived (both are made of vesicular basalt); all other cases are fragments of varying degrees of preservation. The only two complete pieces measured 24 cm x 17 cm x 9 cm (weight 4 kg) and 24 cm x 20 cm x 7 cm (weight 4 kg).

**L4** (n =  27) – these are very similar to the previous types (Fig 19: 3, and Fig 23: C), but they differ in that they are of irregular shape (O4), which is not the case for L3, and they do not have such high rims (LS4; TS2). However, they resemble the previous subtype in length and width. Almost half of the items (n =  12; 44.4%) were preserved in a complete state. They feature up to 10 different raw materials, including very rare conglomerate (with a modelled “hand motif” on its dorsal part; Fig 19: 3). However, the most common raw material is again vesicular basalt (44.44%). The complete smallest piece measures 10 x 8 x 4 cm, the largest 38 x 20 x 11 cm. The weight varies between 0.3 kg and 12.5 kg (Median =  2.59 kg).

**L5** (n =  17) – are simple tools of mostly bolder form, of variable metrics, without detected shape modification. LS and TS are typically straight (LS1; TS1). The representation of raw materials is variable.

### Possible U-subtypes (compare Fig 9)

**U1** (n =  1) – the single example has a regular round shape (O1) with a convex LS (LS3) and convex TS (TS2). We assume that the U1-subtype was moved in a circular motion during use, similar to a pestle. Measuring 15 cm length (weight is 1.7 kg), this single piece, made of gabbro, was used with only one hand, or with the hands placed above each other.

**U2** (n =  39) – except for the round shape (O1), all other shapes occur in this subtype. The LS is always convex (LS3), and TS is also characteristically convex (TS2). This subtype was moved in back-and-forth motion during use, paired with a lower stone with an elongated concave active surface. Up to 58.97% of these subtypes are made of vesicular basalt. The representation of other raw materials is varied, but none is represented by more than 5 pieces. The smallest complete piece measures 16 x 15 x 4 cm, the largest 34 x 19 x 6 cm. The weight is between 1.4 kg to 8 kg (Median =  2.9 kg).

**U3** (n =  34) – occurs exclusively in regular carefully manufactured shapes: O1-O4; Q6 (Fig 20: 1-2). The LS is typically, but only slightly, concave (LS2). The TS is almost always straight (TS1) or only slightly convex. Most often, this subtype was used in a back-and-forth motion. Vesicular basalt represents a total of 64.71% of the determined raw materials. The representation of other raw materials is similar to the previous subtype. The smallest complete piece measures 15 x 13 x 6 cm, the largest 33 x 18 x 8 cm. The weight is between 0.95 kg and 7.5 kg (Median =  2.4 kg).

**U4** (n =  252) – encompasses artifacts of all different shapes (Fig 20: 3, and Fig 24). However, the LS is always typically straight (LS1), as is the TS (TS1). This is by far the most numerous subtype, again most often made from vesicular basalt (58.73%). Sandstone (ophiolite), dacite, andesite, and rhyolite are also represented in the material composition in more than 4% of pieves (over 10 pieces). The smallest complete piece measures 13 x 9 x 5 cm, the largest 35 x 19 x 6 cm. The weight varies between 0.44 kg and 8.5 kg (Median =  2.6 kg).

**Fig 21:**
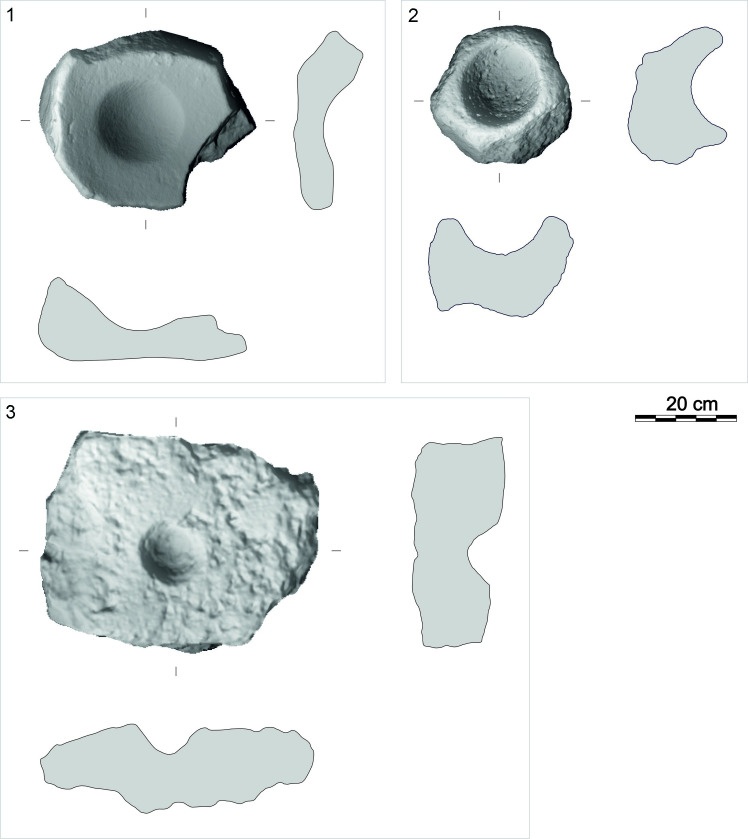
M-subtypes of GMT-class from various contexts. 1: LM-subtype (compact basalt; GK10_67; without recorded context); 2: M1-subtype (vesicular basalt; GK08_157; lower settlement, group C); 3: M2-subtype (vesicular basalt; GK08_48; upper settlement, group F). The abbreviation GK is the designation of the site, the following two digits indicate the year of the excavation, the last digits are the inventory number of the find. For 3D models see S2 Fig. Author D. Pilař.

UL (n =  4) – combinations of lower and upper stones are noted. They do not deviate from the previous subtypes in terms of shape, size or material.

### Possible M-subtypes (compare Fig 10)

M-types can be divided into two basic shape types, differentiated by the location of the active surface (F1 or F2) and especially by how they were manufactured:

**M**1**** (n =  58) – the first group includes regularly shaped mortars (O1-O2; Fig 21: 2, and Fig 25), sometimes finely made by grinding (typical for ignimbrite tools), with the active surface occupying almost the entire work part of the tool (F1). The distribution of raw materials corresponds to the mortar described above. The smallest complete piece measures 12 x 12 x 6 cm, the largest 53 x 39 x 16 cm. Weights vary between 0.8 kg and 56 kg (Median =  15.5 kg).

**M2** (n =  20) – mortars in the second group typically have an irregular body (O3; Fig 21: 3), they are mostly coarsely flaked and sometimes their active surface is placed asymmetrically on the work part (F2). Vesicular basalt predominates in the raw materials represented (60%; n =  12). The smallest complete piece measures 9 x 9 x 4 cm, the largest 54 x 51 x 17 cm. Weights vary between 0.15 kg and 45 kg (Median =  2 kg).

**LM** (n =  5) – are a combination of M-type with L-type (Fig 21: 1), whether on one side or on two opposite sides. The raw materials and sizes are variable.

### Use-wear traces on subtypes

Eighteen artifacts of the GMT-class were selected for specialized use-wear study. The artifacts are mostly made of vesicular basalt which has a hard structure covered by medium to large-sized vesicles (pores) with sharp edges. However, other raw materials were also investigated: soft, porous dacite; tuff and ignimbrite; coherent, hard granite; incoherent, fine-grained sandstone; and conglomerate.

**Fig 22:**
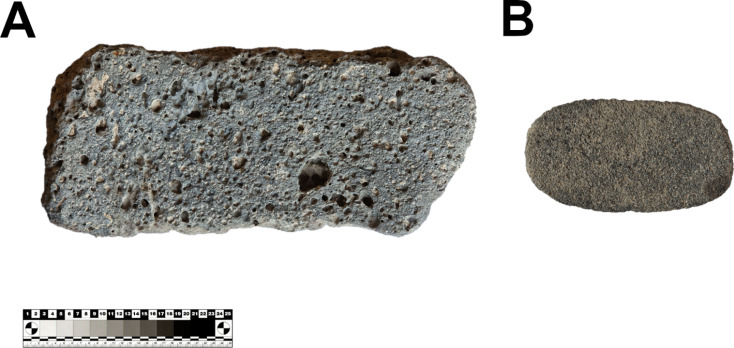
Unused tools. A: L1-subtype (vesicular basalt; GK98_1; no context recorded); B: U3-subtype (vesicular basalt; GK00_112; lower settlement, group A). The abbreviation GK is the designation of the site, the following two digits indicate the year of the excavation, the last digits are the inventory number of the find. Author K. Doležalová.

**Fig 23:**
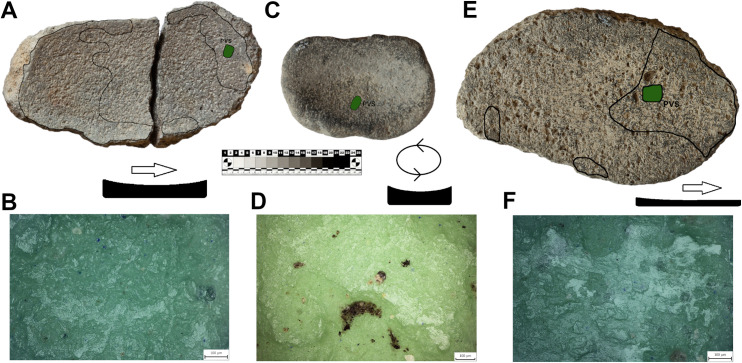
The use-wear analysis of L-types, direction of movement and the development of micropolish. A: L2-subtype (GK01_103; upper settlement, group F) of dacite with marked homogeneous zones and location of silicon cast (PVS), cross section with marked direction of longitudinal movement towards the user; B: The smooth micropolish with domed topography was distributed on the surface in loose connected large patches, 200 × magnification; C: L4-subtype of vesicular basalt (GK99_91; lower settlement, group A) partly covered by calcareous crust with marked location of silicon cast (PVS), cross section with marked circular movement; D: Large patches of smooth, domed micropolish with short and wide striations, 200 × magnification; E: L1-subtype of vesicular basalt (GK99_132; lower settlement, group A) partly covered by calcareous crust with marked homogeneous zones and location of silicon cast (PVS), cross section with marked direction of longitudinal movement towards the user; F: Large patches of smooth micropolish with domed to flat topography and without any linear traces, 200 × magnification. The abbreviation GK is the designation of the site, the following two digits indicate the year of the excavation, the last digits are the inventory number of the find. Author K. Doležalová.

Seven of the eight L-types examined using use-wear analysis show clear use-wear traces associated with grinding-milling activities in the form of levelled surfaces, scratches and rounding of mineral grains. Surprisingly one of the L1-subtypes (Fig 22: A), which is clearly a completely finished tool, was never used for any kind of operations (at least for processing of cereals). The rest of the lower stones were made to different designs and were used in various ways (longitudinal/chaotic movements) for grinding plants. However, only three L-subtypes bear well-preserved wear patterns that could possibly be connected to grinding of cereals (Fig 23). On their surfaces we observe large levelled areas (homogeneous zones), where the mineral grains are levelled, have rounded edges and are covered by microscopic smooth, domed polish, which is distributed on the surface in loose connected large patches.

**Fig 24:**
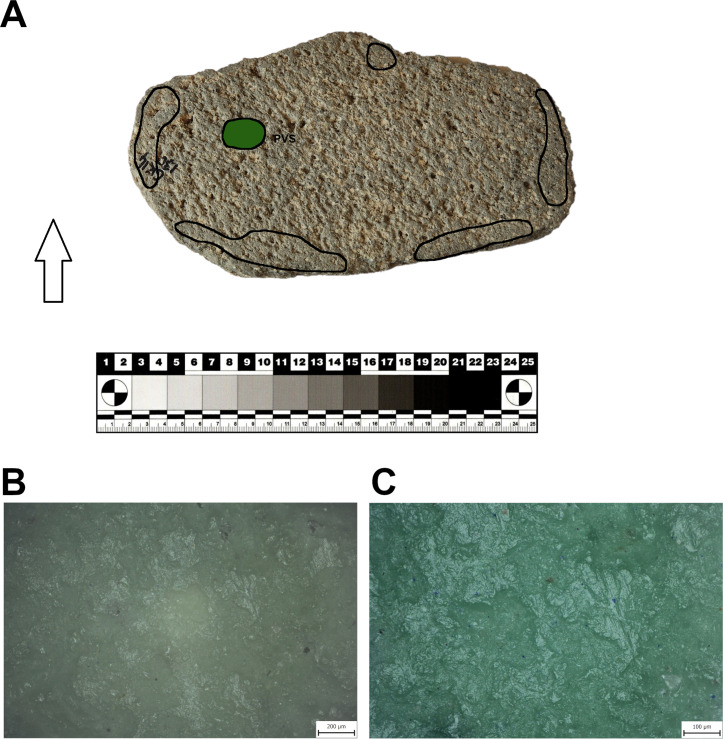
The use-wear analysis of upper stone. A: U4-subtype of dacite (GK14_130; lower settlement, group E) with marked homogeneous zones and location of silicon cast (PVS), the arrow indicates the direction of movement towards the user; B: Loose connected large patches of micropolish on the high topography of the surface, 100 × magnification; C: Smooth, domed micropolish with rarely occurring wide and deep striations, 200 × magnification. The abbreviation GK is the designation of the site, the following two digits indicate the year of the excavation, the last digits are the inventory number of the find. Author K. Doležalová.

The eight examined U-subtypes show different degrees of use. Except for one U3-subtype (Fig 22: B), which was probably never used (the same case as with one of the aforementioned L-subtypes), a total of six U-subtypes show use-wear traces that could be connected to the grinding of plant material (Fig 24).

**Fig 25:**
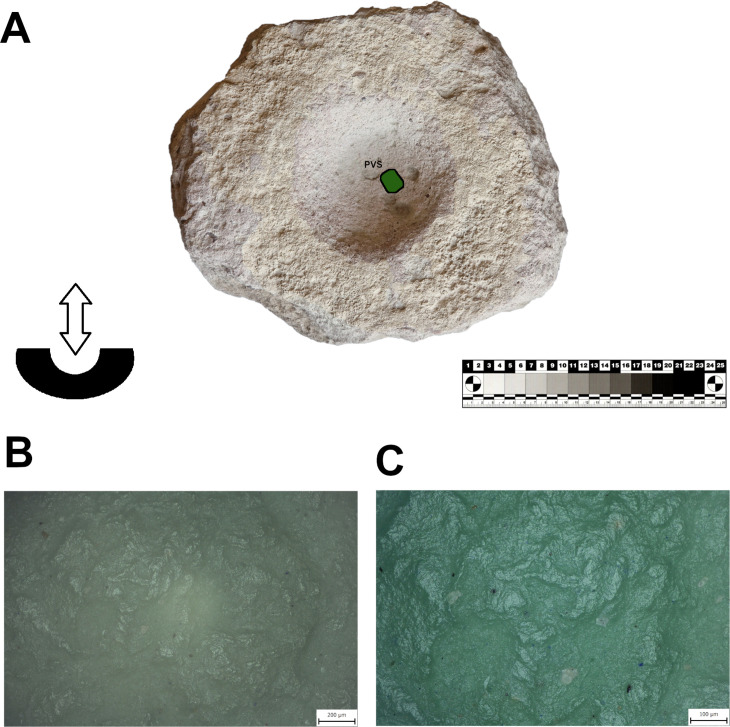
The use-wear analysis of an M1-subtype. A: M1-subtype mortar (GK17_18; lower settlement) of ignimbrite with marked location of silicon cast (PVS), cross section with direction of movement; B: Separate small patches of micropolish proceeding from the higher to the lower part of the topography, 100 × magnification; C: Smooth micropolish with domed to flat topography without any linear traces and the crystals appear to by polished and abraded, 200 × magnification. The abbreviation GK is the designation of the site, the following two digits indicate the year of the excavation, the last digits are the inventory number of the find. Author K. Doležalová.

Only two pieces belonging to the M1-subtype and made of soft raw materials (tuff, ignimbrite) were analyzed for use-wear. Surprisingly, the mortar made of tuff (GK13_24) shows no signs of use-wear traces connected to grinding, pounding or crushing activities, and only traces of its original manufacture were identified. This means that some pieces shaped like this were used only as containers or were still unused when abandoned. On the other hand, another mortar of local ignimbrite (GK17_18; Fig 25) bears well-developed use-wear traces indicating that it was used for processing plant matter. Mineral grains on the sinuous working surface have rounded edges. The domed micropolish covers the high and low topography of the surface and crystals are abraded and polished (Fig 25). However, a pattern indicative of stone-on-stone contact was not identified, so, it is assumed that the active complementary tool of the original mortar-pestle set was made of some other material, probably wood.

We can conclude this informative part with the observation that there is no clear connection between a specific purpose and a specific design or raw material in our selected group of artifacts. Nevertheless, it must be noted that we worked with a small group of tools in this part of the study and not all of the defined subtypes were analyzed. On the other hand, even from the results of this initial analysis of the GMT assemblage we can highlight the fact that besides the tools, M-types probably also include containers, and finished, but unused, complete tools.

### Vessels

**Fig 26:**
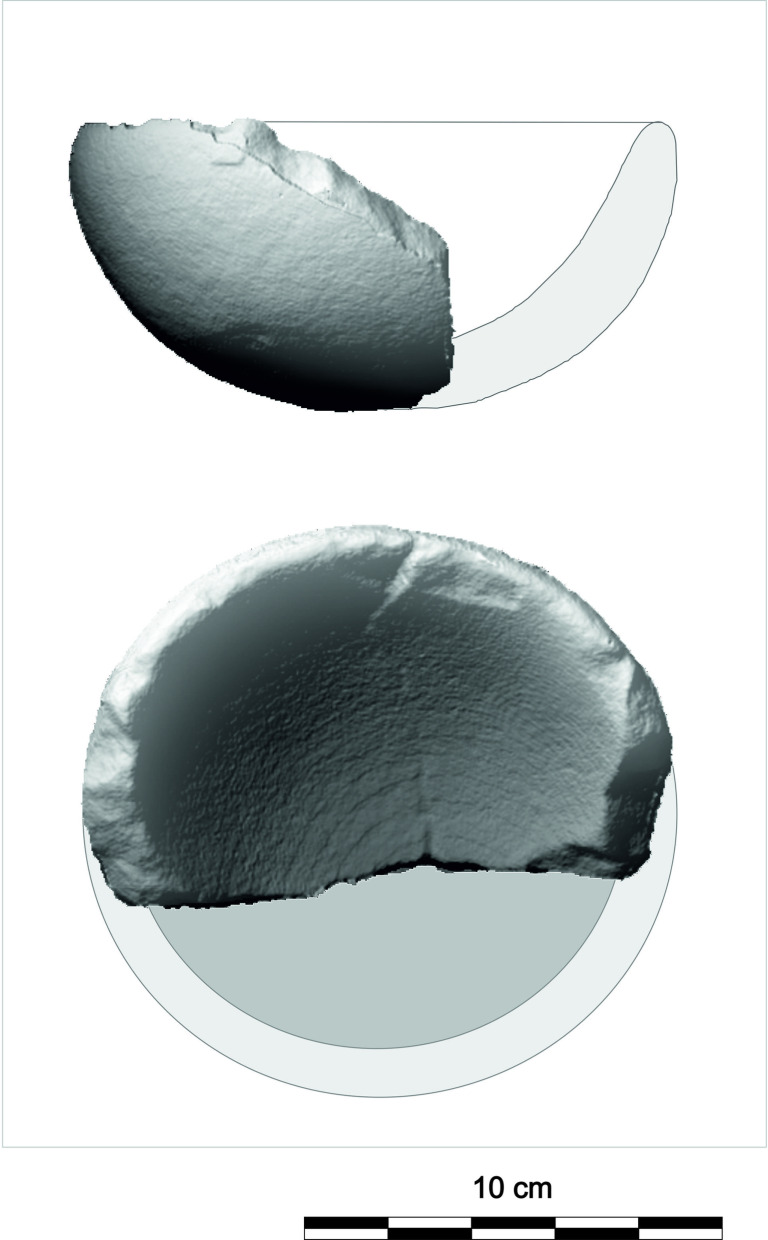
An example (limestone; GK09_119; lower settlement, group E) of a reconstructed V-class fragment. The abbreviation GK is the designation of the site, the following two digits indicate the year of the excavation, the last digits are the inventory number of the find. Author D. Pilař.

All V-class vessels were simple bowl shapes (Fig 26) without any decoration. Various raw materials were used for their production, including marble (n =  3), tuff (n =  3), and less often limestone. While not common finds, these vessels are significant due to the raw materials used, including marble, which is rare in this region.

Only two complete vessels have been preserved. The first measures 22 cm x 15 cm x 10 cm with a weight of 1 kg, the second measures 10 cm x 8 cm x 4 cm with a weight of 0.4 kg. For two other fragmentary pieces, it was possible to reconstruct the original maximum length, i.e., 14 cm and 30 cm. The wall thickness of the 7 measured pieces varies between 1.1 cm and 5 cm.

**Fig 27:**
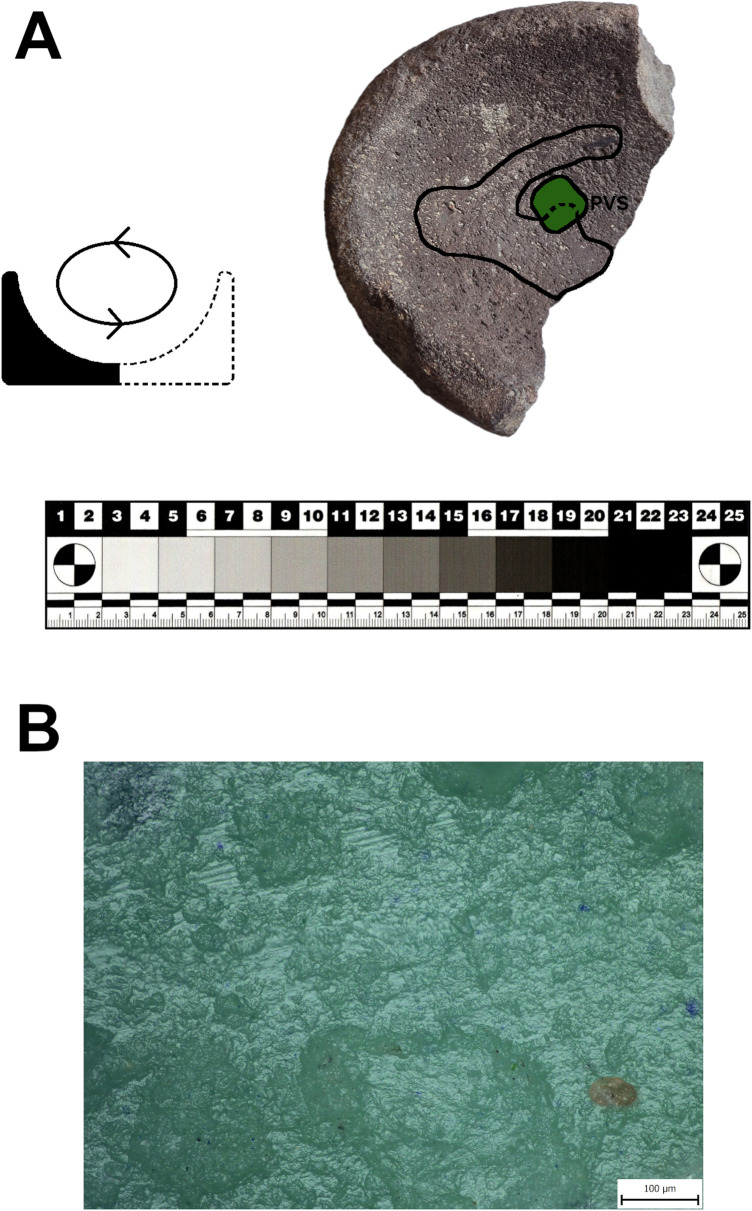
The use-wear analysis of a V-class artifact. A: Vessel (GK96_1; no context recorded) of hard pumice with marked homogeneous zones and location of silicon cast (PVS), cross section with direction of movement; B: Smooth, domed micropolish with long, wide and deep striations is densely distributed on the high topography in large patches, 200 × magnification. The abbreviation GK is the designation of the site, the following two digits indicate the year of the excavation, the last digits are the inventory number of the find. Author K. Doležalová.

One artifact made of pumice was selected for use-wear analysis to understand the function of this class. From macroscopic observation it appears that it may have been used only as a storage container or as a serving dish. However, according to our results, the artifact (Fig 27) was probably used for grinding activity with circular motion. The working surface of the tool was nicely levelled and covered by long striations. The smooth micropolish was distributed in large patches covering the high topography of the surface.

### Abraders

In the GK assemblage, A-class artifacts, which were primarily used for reducing various hard materials, take the form of plates or nodules and are made of tuff (n =  16), sandstone (n =  3), pumice (n =  2), and more rarely vesicular basalt. They vary greatly in size. The maximum length is 5 cm to 32 cm, width 2 cm to 21 cm, thickness 2 cm to 8 cm. The weight also varies from 7 g to 3.3 kg. As already mentioned, sometimes traces of secondary abrading also appear in other groups.

**Fig 28:**
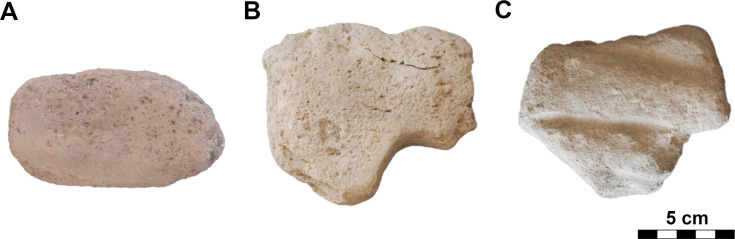
Examples of A-subtypes (abraders). A: A_flat (tuff; GK05_29; upper settlement, P1); B: A_flat (tuff; GK08_119; upper settlement, group F); C: A_groove (tuff; GK07_72; without recorded context). The abbreviation GK is the designation of the site, the following two digits indicate the year of the excavation, the last digits are the inventory number of the find. Photo by J. Řídký.

In the artifacts that were classified as A-class, two macroscopically legible types of use traces appear - grooves and flat surfaces. These are the only characteristics by which it is possible to divide them into two basic subtypes:

**A_groove** (n =  19; Fig 28: C) –their surface bears grooves that vary in length, number (1 – 8 times) and direction (parallel, chaotic). Grooves are U-shaped, with widths of between 7 mm and 13 mm.
**A_flat** – (n =  24; Fig 28: A-B) – are of various shapes. Abraded surfaces appear either on one or several sides.

### Multiple-use tools

The PRT-class is a very diverse group of multiple-use tools in terms of shape and raw materials. As regards the raw materials used, nearly all recorded variants appear here. The vast majority are suitably hard and occur in natural form (mostly nodules – pebbles/cobbles) on the banks of the Melendiz River. The tools can be very small, measuring as little as 2 cm in length, but also much larger with lengths of up to 34 cm (Table 5). However, tools with a maximum length of 7 cm to 11 cm, a width of 5 cm to 8 cm and a thickness of 3 cm to 6 cm clearly prevail. The weight usually varies between 0.15 kg and 0.62 kg, but there are a considerable number of exceptions with a much higher weight, apparently mirroring the variety of raw materials exploited. In general, the vast majority of PRT-class tools could be used with one hand.

**Table 5:** Comparison of length, width, thickness (all in cm) and weight (in kg) of the artifacts of PRT-class.

Cases	Min.	Max.	Mean	Std.Error	Median	Std.Deviation	QI	QIII	IQR	Range
**LENGTH**										
418	2	34	9,2	0,176	9	3,594	7	11	4	32
**WIDTH**										
418	1	24	7,1	0,129	7	2,639	5	8	3	23
**THICKNESS**										
418	1	16	4,71	0,099	4	2,016	3	6	3	15
**WEIGHT**										
418	0,002	17,8	0,582	0,059	0,035	1,2	0,154	0,615	0,461	17,798

QI =  lower quartile (25%); QIII (upper quartile (75%); IQR =  interquartile range. Author J. Řídký.

Macroscopically visible use traces tend to be located (WP; Fig 13) in different parts of artifacts, without a noticeable pattern. Although it can be assumed that they were multifunctional tools, it was possible to macroscopically observe some predominant use traces (WT; Fig 13) on their surfaces – namely, traces resulting from polishing, pounding and coarse hammering.

**Fig 29:**
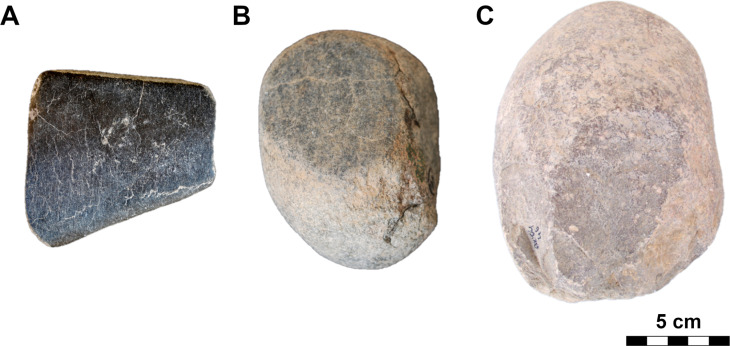
Examples of PRT-subtypes (multiple-use tools). A: PRT_polishing (compact basalt; GK06_124; upper settlement, group F); B: PRT_pounding (quartz; GK08_129; upper settlement, group F); C: PRT_hammering (quartz; GK04_46; upper settlement, P1). The abbreviation GK is the designation of the site, the following two digits indicate the year of the excavation, the last digits are the inventory number of the find. Photo by J. Řídký 2009.

For the purposes of this work, three subtypes have been identified:

**PRT_polishing** (n =  135; Fig 29: A) – are tools of various shapes and dimensions (maximum length is between 2 cm and 34 cm), for which polishing was recorded on various parts of the body.
**PRT_pounding** (n =  110; Fig 29: B) – similar shapes and dimensions (maximum length is between 4 cm and 16 cm), but with traces of fine hammering or/and pounding.
**PRT_hammering** (n =  87; Fig 29: C) – are the heaviest tools by weight (in GK assemblage, up to 3.8 kg; with a length of between 5 cm and 20 cm) among the PRT-class, for which traces of harder hammering were detected. It differs from the traces characteristic of the previous group by the bigger strike scars.

### Drilled items

**Fig 30:**
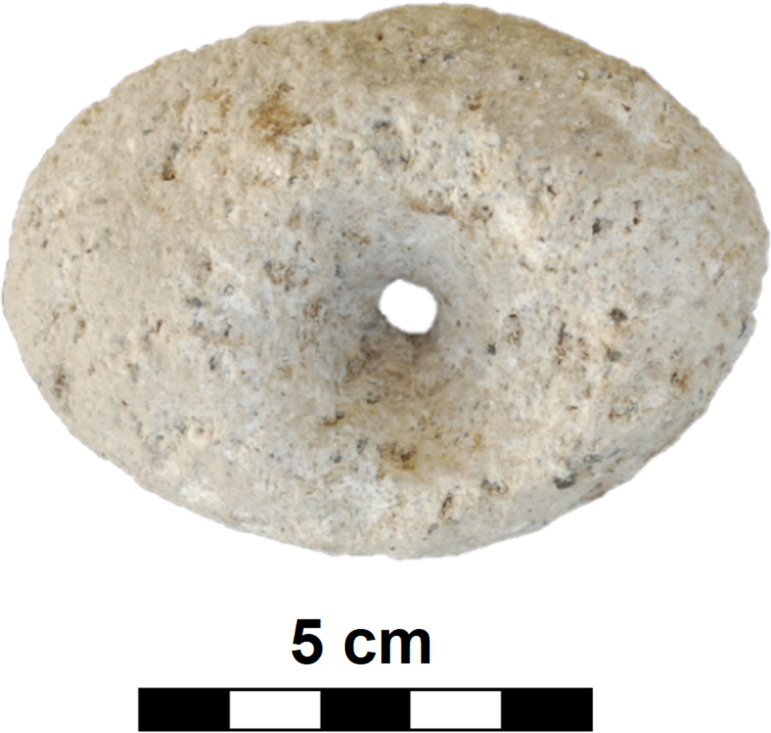
An example of a D-class artifact (drilled items), classified as a textile weight (tuff; GK08_101; upper settlement, P1). The abbreviation GK is the designation of the site, the following two digits indicate the year of the excavation, the last digits are the inventory number of the find. Photo by J. Řídký 2009.

Although this D-class also includes perforated pebbles and enigmatic triangular shaped pieces of unknown purpose (n =  5; maybe textile weights), as well as spindle whorls (n =  2) and apparently other textile weights made of tuff (n =  2; Fig 30), the most typical object is a more or less circular, bi-conically perforated disc with a maximum diameter of 11 cm to 15 cm, a thickness of 6 cm to 9 cm and a weight of 0.51 kg to 2.09 kg. Typically, these discs are made of basalt, surprisingly most often vesicular basalt (n =  19; 59.38%), less often dacite (n =  7), or compact basalt (n =  4); only rarely are other raw materials used. The perforation in the central part is always conical on both sides. The dimensions of the hole (dia2; see Fig 7) range from 1.2 cm to a maximum of 2.5 cm (Median =  2 cm).

**Fig 31:**
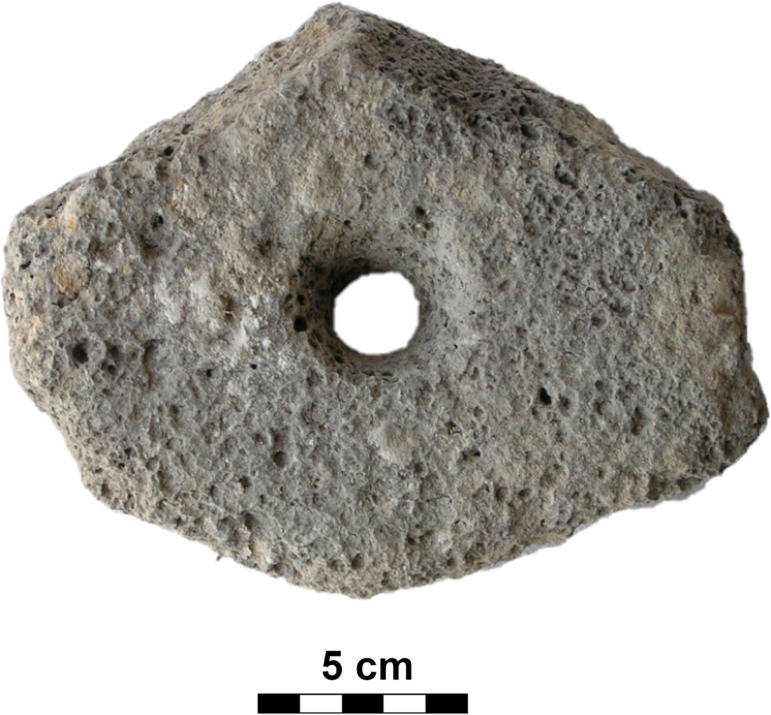
An example of a D-class artifact (drilled items), made from what was originally a U-type (vesicular basalt; GK06_12; upper settlement, group F). The abbreviation GK is the designation of the site, the following two digits indicate the year of the excavation, the last digits are the inventory number of the find. Photo by J. Řídký 2006.

**Fig 32:**
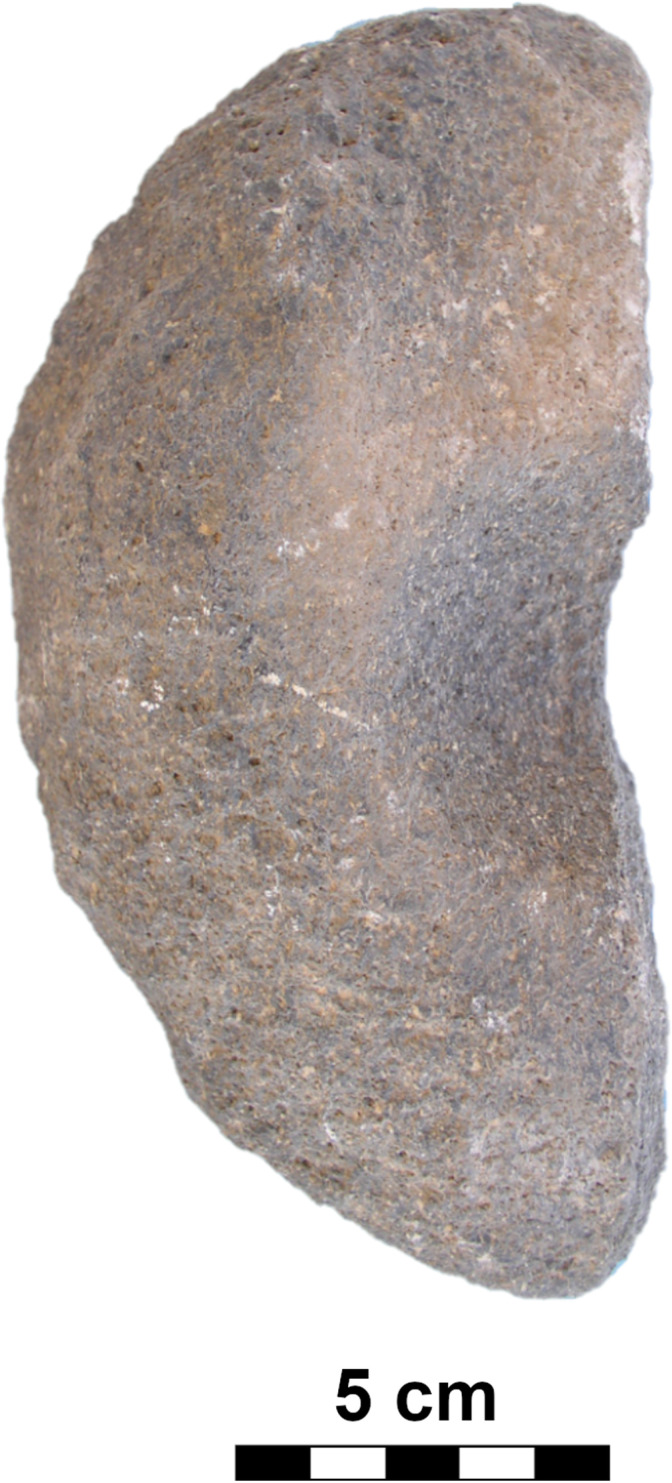
An example of a semi-finished D-class artifact. Without inventory number (vesicular basalt). Photo by J. Řídký 2004.

In at least two cases, D-class artifacts were made from what was originally an upper stone (Fig 31). A total of 60.47% (n =  26) of these artifacts survive as half objects that do not fit to each other. This is surprising especially in the case of vesicular basalt, which is a very hard raw material. Since most of these fragments were flaked during manufacture (n =  21), it is possible that fragmentation occurred during their production. However, pieces with ground surfaces (n =  11) often appear among the D-class. Thus, great care was evidently paid to these items during their production. In a total of 18 pieces (9 of which are complete) the double-sided perforation was not completed (Fig 32).

**Fig 33:**
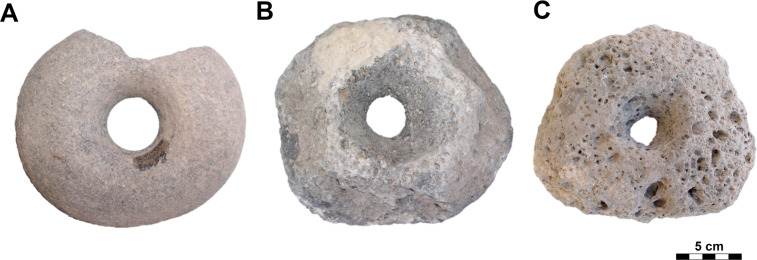
Three examples of D-class artifacts from different contexts. A: ground (compact basalt; GK00_59; lower settlement, group A); B: flaked (without inventory number; vesicular basalt); C: flaked (vesicular basalt; GK99_133; lower settlement, group A). The abbreviation GK is the designation of the site, the following two digits indicate the year of the excavation, the last digits are the inventory number of the find. Photo by J. Řídký 2004.

It is possible to divide this class in three groups: Complete pieces (**D**; n =  14; Fig 33) and fragments (**D_frg**; n =  25), regardless of whether they were flaked or ground, as well as obviously unfinished, i.e., undrilled, pieces (**RD**; n =  18; Fig 32).

### Stone spheres

**Fig 34:**
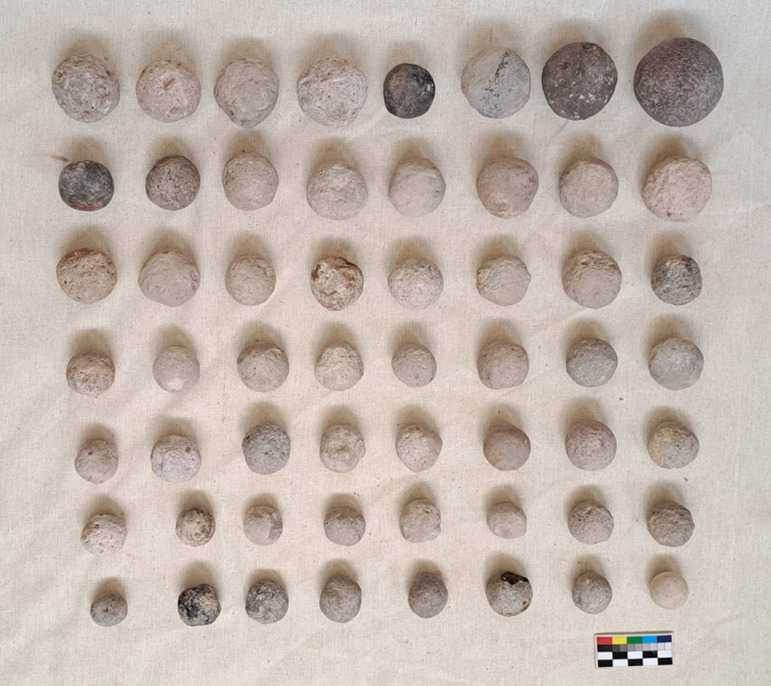
B-class artifacts of various sizes, raw materials and contexts. Photo by D. Pilař 2023.

B-class artifacts are among the most frequent finds at GK (Fig 34). Except for one case that was flaked, all others are ground. For 94 pieces, 20 different raw materials were used for their production, including two cases made of clay (Table 6). These artifacts generally measure 3 cm to 4 cm in diameter (n =  105; 80.77% of 130 complete pieces) and weigh between 20 g and 46 g (Table 7). However, some pieces weigh only 5 g.

**Table 6:** Overview of various raw materials used for B-class artifacts.

Raw material	Ʃ	%
Tuff	24	22.56
Ignimbrite	18	16.92
Basalt compact	11	10.34
Dacite	7	6.58
Limestone	6	5.64
Quartz	5	4.70
Pumice	4	3.76
Rhyolite	4	3.76
Clay	2	1.88
Gabbro	2	1.88
Serpentinite	2	1.88
Amphibolite	1	0.94
Andesite	1	0.94
Diorite	1	0.94
Granite	1	0.94
Green schist	1	0.94
Chert	1	0.94
Obsidian	1	0.94
Sandstone	1	0.94
Silicite	1	0.94
**Ʃ**	**94**	**100.00**

Raw material determination by K. Doležalová.

**Table 7:** Comparison of length (in cm) and weight (in grams) of the B-class artifacts.

Cases	Min.	Max.	Mean	Std.Error	Median	Std.Deviation	QI	QIII	IQR	Range
**LENGTH**										
130	1	8	3,38	0,086	3	0,983	3	4	1	7
**WEIGHT**										
130	5	590	40,64	4,982	30	56,806	19,75	46	26	585

QI =  lower quartile (25%); QIII (upper quartile (75%); IQR =  interquartile range. Author J. Řídký.

Although these objects are commonly identified as sling missiles (e.g., [93,94]; the most common tuff artifacts weigh only 5 g), the range of raw materials used in their manufacture, the similarity in their dimensions and their wide range of colors lead us to suggest that they perhaps served a less utilitarian function. We will come back to this issue in the Discussion section below.

### Plates with dimples

T-class artifacts are enigmatic objects shaped by flaking and most often having a flat oval form. Complete pieces (n =  6) reach a length of 24 cm to 33 cm, a width of 17 cm to 28 cm, a thickness of 4 cm to 7 cm, and a weight of 0.43 kg to 6 kg. They are made from different raw materials, most often rhyolite (n =  5; 50%), less often local ignimbrite or dacite. A complete piece is on display in the Aksaray Museum.

**Fig 35:**
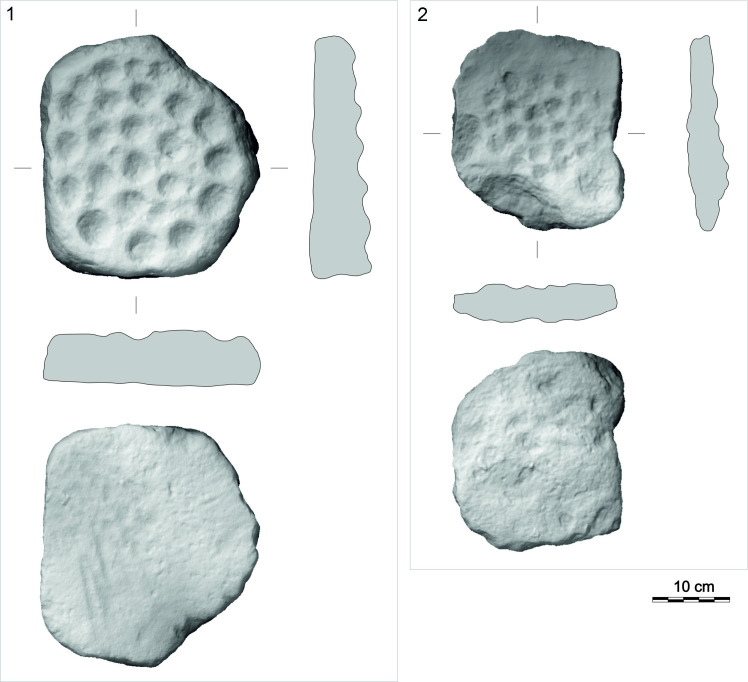
Two examples of T-class artifacts (plates with organized dimples) from different contexts. 1: GK09_36 (rhyolite; without recorded context); 2: GK10_207 (ignimbrite; upper settlement, group F). The abbreviation GK is the designation of the site, the following two digits indicate the year of the excavation, the last digits are the inventory number of the find. For 3D models see S2 Fig. Author D. Pilař.

**Fig 36:**
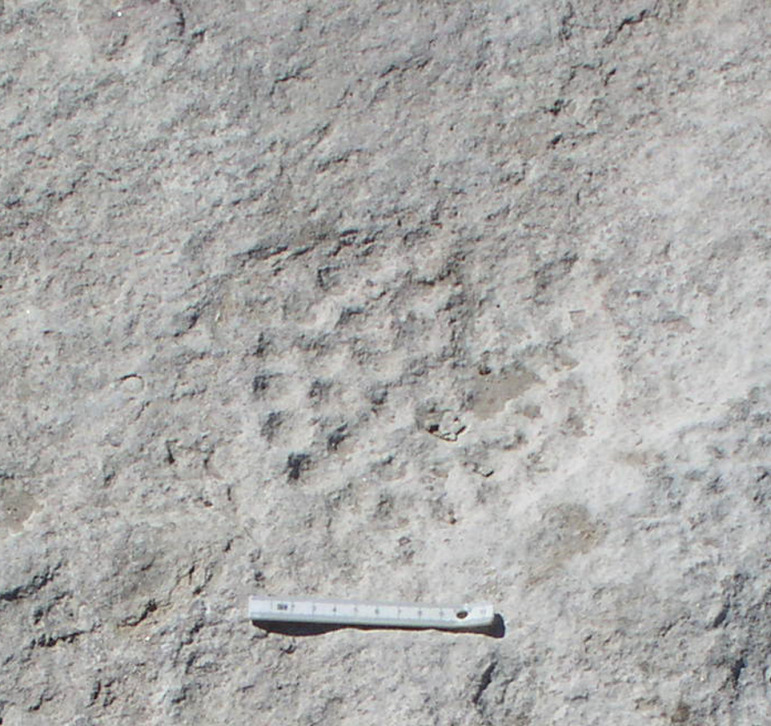
Similarly organized dimples of the same sizes on the surface of the rhyodacite/ignimbrite rock in the area of the Güvercinkayası site (lower settlement). Photo by J. Řídký 2004.

The regular and macroscopically smooth round dimples, with a diameter of around 4-5 cm and a depth of up to 2 cm, are, except for one case, arranged on only one side of the object. The total number of dimples on complete pieces varies from 17 to 23 (Fig 35), however, dimples are always arranged in a circle or spiral. Similarly arranged dimples were also recorded on the surface of the ignimbrite rock on which the settlement was situated (Fig 36). This rather precludes the interpretation of these artifacts as, for example, pallets.

One of the items (GK14_44) was subjected to use-wear analysis to understand the possible purpose of the dimples, but no wear traces connected to use were identified. This artifact, therefore, was not involved in processing of other objects. As with the previous class, we will speculate about the possible purpose of these artifacts in the Discussion section.

### Fragmentation of the MA assemblage

**Fig 37:**
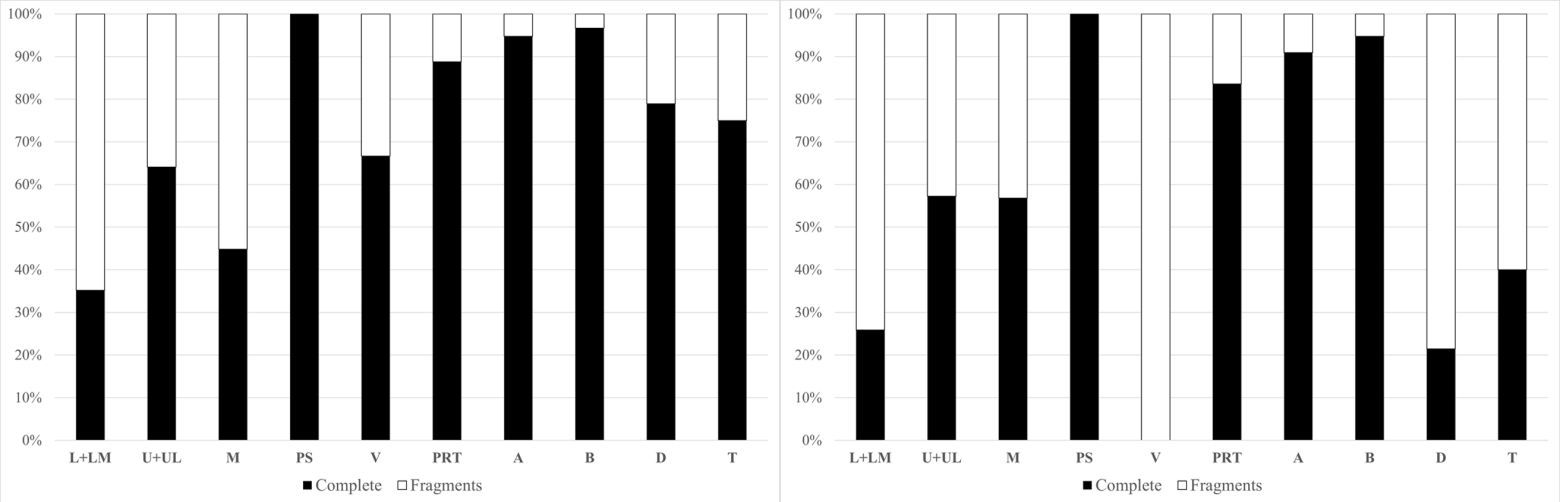
A percentage proportion of complete and fragmented MAs in the upper settlement (left) and lower settlement (right). For abbreviations and amounts see Table 1. Author J. Řídký.

From the comparison of complete pieces and fragments of finished/used artifacts from the entire GK assemblage, it emerges that artifacts in a fragmentary state of preservation mostly belong to the V-class, then L + LM-types of GMT-class, and D-class (Table 1; Fig 37). In contrast, B-class, A-class, and PRT-class, are conspicuously predominant among artifacts in a complete state of preservation. Only PS-types are recorded exclusively in complete condition, but only 10 atypical pieces represent them.

For a total of 390 GMT fragments, approximately what percentage of the original size of the tool was preserved was recorded. According to this rough estimate, the vast majority, that is 319 (81.79%) of finished/used tools, have been preserved between 10% and 50% of their original size. That means that about three-quarters of the fragmented L-types, M-types, and U-types survive at less than half their original size. However, only one single L-type and seven U-types bear traces of deliberate strikes in the utilization areas, which apparently caused their fragmentation. It seems likely that the destruction of most GMTs occurred rather accidentally, during use [95].

### Spatial analysis

#### Lower and upper settlements.

As already mentioned, the GK assemblage contains unfinished items (roughouts/semi-finished products) belonging to two recorded classes (GMT and D-classes) in various stages of manufacture (Table 1). It was possible to attribute 48 pieces to either the lower or upper settlement (Table 8 and Fig 1). It is clear from their distribution that roughouts/semi-finished products of all recognizable artifact forms occur in both parts of the settlement (S2 Table). Also, both settlements are very similar in terms of the overall composition of finished/used MA classes and types. The only difference is the higher representation of the PRT-class in the upper settlement. In a subsequent step, MA subtypes were compared between the lower and upper settlements. In this case, however, the only exception is the more frequent occurrence of PRT-subtypes, bearing traces of harder hammering (PRT_hammering), in the upper settlement (S3 Table).

**Table 8:** Overview of roughouts/semi-finished products from upper and lower settlements.

	Upper Settlement				Lower Settlement			
Type Or Class	Total	%	Ʃ Complete	% Complete	Total	%	Ʃ Complete	% Complete
Roughouts of L (RL)	3	15.79	2	16.67	1	3.45	1	5.56
Roughouts of U (RU)	9	43.37	6	50.00	16	55.17	13	72.22
Roughouts of indeterminable GMT (R)	0	0.00	0	0.00	2	6.90	0	0.00
Roughouts of M (RM)	1	5.26	1	8.33	1	3.45	0	0.00
Roughouts of drilled items (RD)	6	31.58	3	25.00	9	31.03	4	22.22
Ʃ	19	100.00	12	100.00	29	100.00	18	100.00

Author J. Řídký.

In comparing the MA metric characteristics between the lower and upper settlements, only L-type, U-type, and PRT-class could be considered because other groups were not sufficiently represented (n =  30 was set as a threshold). L-types in the upper settlement turned out to be on average 2 kg heavier, and were generally larger, than those in the lower settlement. Greater robustness was also clear in the case of the PRT-class, which were 1 kg heavier in the upper settlement (S4 Table). However, the majority of these differences were not found to be significant during statistical testing, mainly due to the high variability of both L-types and PRT-classes. The only statistically significant difference is the greater thickness of the PRTs in the upper settlement.

Comparison of the metric variables of the MA-subtypes (n =  10 was set as threshold) in the lower and upper settlements mostly mirrors the above observations (S5 Table). The L1-subtype was overall more robust in the upper settlement, with width clearly emerging as a statistically significant value. Likewise, in the PRT-class, the PRT_pounding subtype was noticeably more robust in the upper settlement. The width and weight values proved to be statistically significant (the difference in weight was 0.2 kg). In contrast, all defined U-subtypes were extremely homogeneous in terms of metric variables.

#### Smaller settlement units.

In the next phase of the analysis, the MA composition was compared for individual spatial units (Fig 3). The frequency of finished/used MAs is very variable and for most spatial units we do not have a representative data sample (S6 Table). Therefore, only house groups A, C, E, F, and G, were included in the analysis, followed by corridors R3, R4, R5 and the entire area of P1 (fortification) (S7 Table), and tests for differences in metric characteristics were carried out only for the mentioned groups of houses.

As regards the occurrence of more numerous roughouts of L-type (RL) and U-type (RU), or roughouts of D-class (RD) in smaller spatial units, there is no significant pattern visible. However, the results show some differences in the overall composition of the basic MA classes and types. In this regard, the high representation of PRT-class in house group F and in the adjacent group P1, is noteworthy. Another distinctive pattern is the low representation of B-class in group A houses.

**Fig 38:**
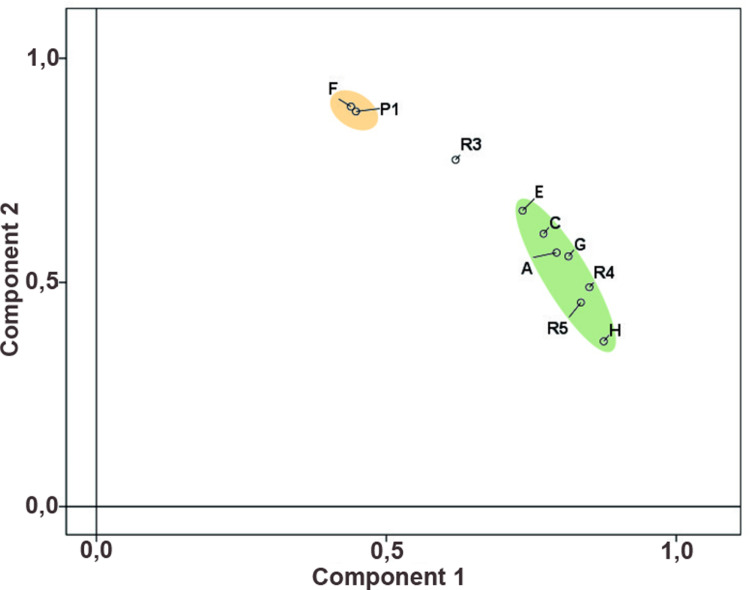
PCA results showing a similar composition of MA types in units around the fortification (yellow) and other groups (green). Author D. Pilař.

**Fig 39:**
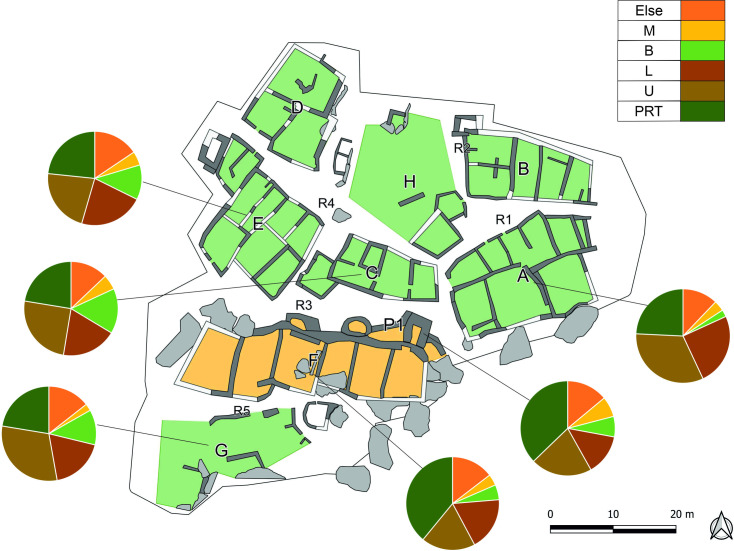
Share of the most numerous classes (B and PRT) and types (L, M, and U) in representative units. Author D. Pilař.

Subsequently, factor analysis of the frequency of MA classes and types in individual groups was performed, with the aim of identifying possible patterns (S8 Table). During factor analysis, spatial groups were extracted into two components with similar factor loading (Fig 38). Groups in individual components are also spatially adjacent to each other (Fig 39). An important finding is that, if we subtract the mentioned overrepresented PRT-class, there is a very balanced occurrence of L-types and U-types in all smaller spatial units.

The comparison of metric variables of MA classes and types in individual units reveals few differences (S9 Table). The PRT-class was generally more robust in the F group, which is illustrated by the significantly higher thickness value. PRT-class is also heavier in F and G groups than in the others, but due to the high variance of the values, this difference is not statistically significant. A second significant difference was found in the lengths of the U-types in group C houses, where, despite the extraordinary metric uniformity of this type, the artifacts were on average 3 cm shorter.

**Fig 40:**
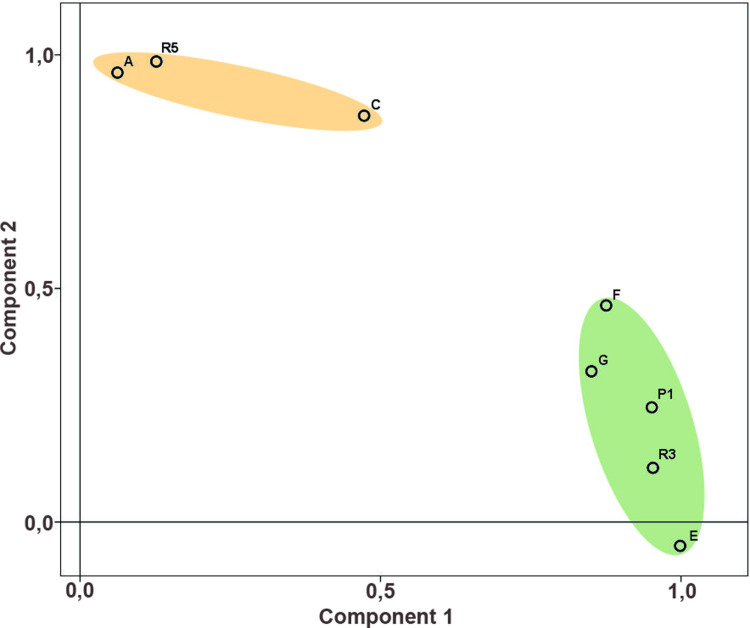
PCA results showing a similar composition of L1-subtypes in groups A, C, R5 (yellow) and groups E, F, G, P1, R3 (green). Author D. Pilař.

**Fig 41:**
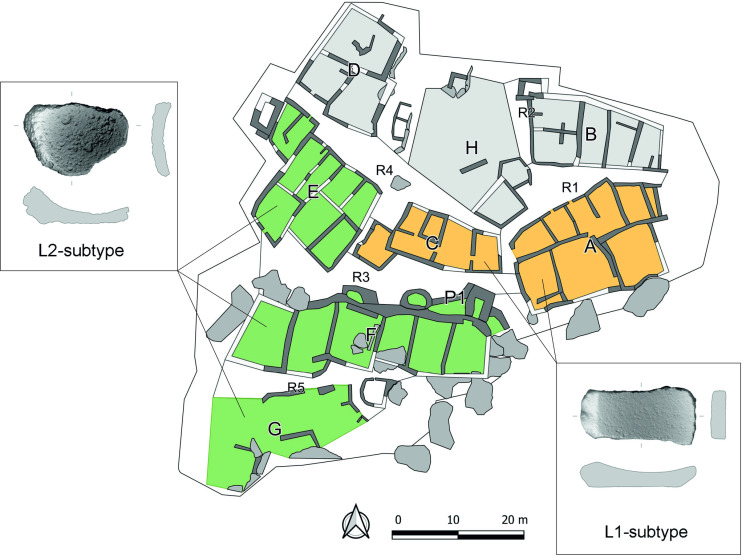
Distribution of the most numerous subtypes (L1 and L2), typically occurring in some units. Author D. Pilař.

In the next step, the representation of individual subtypes in the spatial groups was compared. The subtypes defined in the L-types and PRT-classes (S10 Table) were shown to be variable between individual units. In contrast, variability was absolutely minimal in the numerous group of U-subtypes. In the case of L-subtypes, one can mainly observe the different distribution of subtypes L1 and L2. Using factor analysis, two clusters were found among the spatial groups, which have a similar factor load (S11 Table; Fig 40). In the case of both clusters, most groups are spatially adjacent. If we accept the established premises, we can assume that communities living in the groups of houses described as A and C, generally preferred different shapes of lower stones for food processing than in the rest of the settlement (Fig 41).

Further variability was observed in the distribution of subtypes in the PRT-class. There are numerous differences in the representation of tools used for hammering, polishing, pounding, or for unspecified use. The distribution of these subtypes does not, at first sight, show any structure, and therefore the data were analyzed using factor analysis. During this step, three components were extracted, which represent most of the variability of the data (S12 Table). On an interpretive level, it can only be stated that the activities connected with the use of PRT-class were very diverse in different areas of the settlement. The exception is again group F houses, where all PRT-class subtypes are numerously represented.

Classes, types, and subtypes with lower representation occur in various units around the entire settlement (see Fig 3). Despite that, for example, V-class artifacts were not confirmed in spatial groups C and A, but one find comes from R1 (a corridor between A and C). The same can be said about the presence of T-class (single pieces in all groups of houses, except B and H, but present in corridor R4 between B and H), or B-class (absent in group B houses, but present in corridor R1 in close proximity) or A- class (valid for both subtypes), and D-class.

## Discussion

In what follows, we will first look at how the composition of the MAs changed (if at all) compared to the preceding Neolithic period in western Cappadocia (today’s part of Central Anatolia; [7,96,97]), and then we will test what the composition and distribution of the classified MA groups in the MCH assemblage tell us about the activities in different parts of the settlement and possible social structure.

If we leave aside the S-class artifacts (“portable door sockets”), which were architectural elements, and the class marked as “OTHER” (several “binding stones” and individual items of unknown purpose), there remain at least seven classes that can, to a great extent, be compared to artifacts from the previous period in Cappadocia. All the main MA groups, such as GMT-class (with L-types, U-types, M-types, and PS-types), A-class, PRT-class, but also D-class, B-class, AXE-class, and V-class, have more less similar dimensions to Neolithic artifacts present in the GK assemblage [7,96,97]. The only different and new MA group is the one that we term as T-class (see below).

A closer look at the details reveals differences in overall proportions. Already at the beginning of this work, we pointed out that, for example, cutting edge tools (mostly fragmented axes/adzes; altogether 8 cases, including the pieces in Aksaray Museum) make up only a marginal part of this MCH assemblage. Could it be that these were such valued tools that they received increased attention during the abandonment of the settlement? The method of abandonment of this particular settlement has not yet been completely resolved, but for example I. Pavlů [16] has previously drawn attention to the MAs on the floors of the rooms; it has been suggested that they were transferred to the rooms for various reasons, for example when the settlement was abandoned. However, other tools and other items, such as ceramic bowls and jars seem to have remained approximately where they were used or stored. Furthermore, the presence of GMT roughouts (whole pieces of both, lower and upper stones) and completely finished, but unused tools (complete pieces, confirmed by use-wear analysis) is also pointing to more or less original locations of daily used items. We therefore do not consider this explanation likely. At the MCH site of Yumuktepe-Mersin, where the settlement area is similarly divided by a fortification wall (Level XVI; [29,70]), polished cutting tools were also rare. Could cutting tools have been made from other materials, such as copper [29], or were they not used at all at this time? The two copper axes/adzes found at Yumuktepe-Mersin were used for cutting [98], but their presence is not confirmed at GK. Although most of the buildings at GK were built of stone (e.g., [4]), it is hard to imagine that such universal tools were not used, for example, for building roofs or making various objects from organic materials. It is also possible that both sites represent a special settlement type (complex villages; [99]) within MCH settlement patterns and that the mentioned tools could have been used for activities that took place somewhere outside of the study area investigated by archaeologists. Nevertheless, the low occurrence of stone cutting tools (AXE-class) is so striking that it can be considered significant for the given period. The occurrence of another artifact, V-class, is also very low (up to 15 mostly fragmentary cases, including the pieces in Aksaray Museum), and they lack any decoration.

**Fig 42:**
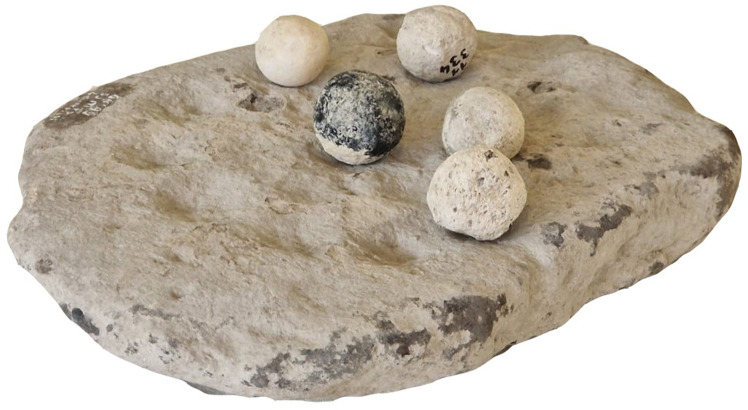
Possible use of B-class (stone spheres) and T-class (plates with dimples) together. Photo by D. Pilař 2023.

We will now focus on a group that as far as we know does not appear in the Neolithic of Central Anatolia, or in the wider area of Neolithic Anatolia. These are artifacts of T-class, plates bearing groups of organized circular shaped dimples (their diameter is around 3-4 cm, depth up to 2 cm). Based on the use-wear analysis, we know that these were probably not tools, at least not tools for processing. But what could they have been used for? There are actually two particularly interesting classes within the GK assemblage that could be functionally related - T-class and B-class. A significant share is made up of more or less regular, completely preserved stone spheres (B-class), with a diameter of 3-4 cm (n = 110; 80% of all B-class). These are made from different raw materials and therefore display a variety of weights, including extremely light examples weighing around 5g; they also come in different colors. The use of certain raw materials, for example compact basalt, or amphibolite, for creating B-class artifacts, required a certain amount of effort and time, more effort than can be imagined for the production of simple sling missiles (bullets), which they are usually considered to be (e.g., [93]; sling missiles are made of clay and are modelled into an oval or bi-conical shape in Yumuktepe-Mersin; [29]). Even though we interpreted the spheres as sling missiles in a previous work [80], based on the study of this larger number of finds we are now leaning towards a different explanation. Although no reliable “in situ” find context was recorded that would directly connect these two classes, we argue that both classes could be functionally related. The sizes of B-class artifacts and the dimensions of the dimples on the surface of T-class simply fit together (Fig 42). However, we can only speculate as to what their function might have been. It could theoretically be some sort of counting aid, or (more likely) some form of board game (e.g., [100]).

Artifacts of GMT-class also underwent a certain transformation compared to the Neolithic of western Cappadocia [7,96,97]. There is a very low proportion of PS-types, for example, compared to M-types in the assemblage. The artifacts that we classify as pestles in the GK assemblage were not shaped with much effort, compared to previous pestles belonging to the Neolithic period in the region [7,97]. Stone pestles obviously do not belong to the typical tools of the MCH period. The other component of mortar-pestle sets, mortars, are found quite often at the site, mostly in a complete state of preservation, and it is therefore possible that the pestles compatible with them were made from organic materials. This hypothesis is further supported by the results of use-wear analyses of one of the mortars which lacks use-wear indicative of stone-on-stone contact. Ideally a much more detailed comparison between Neolithic and Chalcolithic MAs is required. At the moment, however, there is a lack of publications on other MCH assemblages and such detailed comparison goes far beyond the scope of this work, which comprehensively presents the very first MCH assemblage in Anatolia.

In the study we also tried to determine the functional use through use-wear analysis carried out on several U-subtypes with different shapes. However, no significant differences could be demonstrated in our tested group. Statistical analysis of metric properties and raw materials showed high uniformity among these tools. It is therefore possible that shape modifications of U-subtypes are the result of individual user (individual household tradition) preferences, for example for ergonomic reasons.

Now we will focus on the composition and distribution of MAs in individual spatial units and what it may or may not signal about the activity areas and social structure of the GK site. There are a number of gaps in our knowledge of the MCH in Central Anatolia (ca 5,500 – 4,250 cal BCE), the most serious of which is probably the lack of publications on different types of sites and detailed intrasite comparisons (e.g., [99]). In short, we cannot compare our findings with other MCH assemblages from similar or different settlement types. In general, according to current knowledge, most known layouts of MCH settlements in Central Anatolia: “reflect older traditions of the plain” [13]. Although a number of cultural influences from western Anatolia, the Balkan Peninsula, and from the south-eastern direction meet in Central Anatolia, continuity of older traditions is still evident here in settlement layouts [15]. In terms of burial customs, however, even though we lack more detailed information about the given period, we observe a certain shift in the locations of burials and the places where males and females were buried (e.g., [101]).

Arguments that some kind of elite lived in the fortified upper part of the GK site can be based on three observations – the houses in the upper settlement (group F) have greater storage capacity, they are larger in size, and finds of animal bones suggest the consumption of high quality meat [4,5]. It seems that while the lower settlement yields various remains of animal skeletons pointing to the butchering of animals, the upper settlement more often yields bones attesting to higher quality meat [4,37]. It would be interesting to verify if better quality pieces of meat in the upper settlement had been smoked (e.g., [102]). It is one of the many ways of storing, and may have been an activity that was concentrated in a certain phase of the protected upper settlement.

We can present a somewhat different picture based on the results of our comprehensive study of MAs. At first glance, MAs in the upper and lower settlements do not differ in any significant way, neither in the occurrence of morphometric MA groups, nor in their state of preservation. Slight differences can be observed, however: 1) in the occurrence of more robust L-types of GMT-class and PRT-classes (bearing traces after hammering) in the upper settlement; 2) in the occurrence of specific forms of L-types in A and C house groups (both in the lower settlement). These findings could indicate diverse activities in individual households, but, at the same time, we cannot rule out individual tendencies towards variations in the processing of the same products. This interpretation model must be further tested by much more comprehensive use-wear analyses and analyses of other finds. For now, none of the results of analyses of particular morphometric MA groups directly prove the presence or, on the contrary, the conspicuous absence of certain activities associated with MAs in any part of the excavated settlement area (lower and upper settlements).

Does the occurrence of stone raw materials show any distinct patterns proving differential access by individual units/groups of houses or by lower/upper settlements to the resources (e.g., [24])? Many MAs were made of local raw materials, which encompass pebbles/cobbles gathered from the riverbank (gabbro, compact basalt, quartz, etc.) of the Melendiz River and rocks from nearby volcanic outcrops (ignimbrite, tuff and pumice): the largest number of raw materials probably come from regional sources. These are mainly vesicular basalt and sedimentary rocks (favoured for GMT production), sourced within at least 10 km of the settlement. The only supra-regionally sourced raw material (more than 20km away) was marble and probably green schist and metabasite, the exact source of which is unknown. However, original cutting edge tools made of green schist and metabasite have been found mostly in secondary use as hammer stones (PRT-class) in different spatial units. All the mentioned raw materials come from the lower and upper settlements and from different smaller units of the settlement. Marble occurs in about six cases, but even here no significant distribution to certain parts of the settlement can be confirmed.

Our results are based on statistical analyzes of the entire MA assemblage. However, differences between the parts of the settlement may exist in detail, namely in the types of food processed and consumed. We are of course aware that other analytical approaches must follow; these should focus on use-wear based on experiments, and a more detailed analysis is needed regarding the range of utilization areas [103]. Spatial comparisons with other categories of finds are still lacking, unfortunately including archaeobotanical remains which are important for foodway studies connected with GMTs. In summary, based on the composition and distribution of MAs at the GK site, we lean towards the same explanation as that forwarded for the contemporary MCH site of Yumuktepe-Mersin in south Türkiye [29]. In short, the fortification wall protected an area where certain activities took place, including storage, rather than strictly defining areas inhabited by certain social groups, as we observe in later periods (e.g., [104]).

## Conclusion

The assemblage of 1793 macrolithic artifacts, weighing more than half a ton, retrieved from an area of 3,600 m^2^ of the Middle Chalcolithic Güvercinkayası site can be divided into ten classes according to the method of manufacture, raw materials, shapes and dimensions, and functional use. Differences with the previous Neolithic period in the same region are mainly observable in the details, namely low proportions of axes/adzes, stone vessels and pestles. In addition, not too much effort was devoted to the production of pestles.

Most of the spectrum of this category of artifacts remains roughly the same as in the western Cappadocian Neolithic [7,96,97]. An exception is the appearance of a novel artifact type, namely stone plates with arrangements of dimples, which, based on the dimensions of the dimples and the absence of use-wear traces inside the area of dimples, we can associate with similarly 3-4 cm sized stone spheres made of various materials. We speculate that together these objects could be some sort of counting tool or a game (Fig 42). Apart from this last shape class, the shape representation of all other classes is practically the same as in the Yumuktepe-Mersin settlement of the same date in the Mediterranean region of southern Türkiye, which was also divided by a fortification wall [70]. However, the fundamental difference between the two sites, separated by a distance about 200km as the crow flies, is in the lithic raw material used and the low number of MAs found in Yumuktepe-Mersin. In this southern site, mainly rocks of sedimentary origin were used for MA production.

The dimensions of the upper and lower stones of grinding-milling tools in the GK assemblage are so constant that they could have been mass-produced tools; moreover they are mostly made from the vesicular form of regional basalt. We cannot yet say whether the slight differences in the shapes of the lower stones were the result of different functional uses. However, in our opinion, the shapes of the upper stones were adapted for ergonomic or individual household tradition reasons, regardless of the product being processed. Due to the surface sizes of both parts of the upper-lower stone sets, the intention was to process as quickly as possible the largest amount of ground/milled product, probably wheat, barley and certain legumes. The assemblage includes both finished or long-used products and their roughouts/semi-finished products. A significant number of the macrolithic artifacts remained in the place where they had probably been used (on the roofs and/or inside the buildings) or stored. Among the objects are both fragments and whole pieces, which in some cases had never even been used.

The degree of preservation, the raw materials used and the composition of the artifact groups in the individual smaller units of the settlement is almost the same. The same is true when we compare the upper and lower settlements. Based on the study of macrolithic artifacts, it seems that all households represented by groups of houses had the same level of access to objects of any raw material in the settlement and the artifacts were used for similar activities (grinding-milling, pounding, polishing, hammering, and abrading). When we consider the distribution of artifacts from the point of view of social status, it emerges that the Middle Chalcolithic site of Güvercinkayası was not home to a strictly hierarchical society such as those known from social/cultural anthropology [105–112]. Considering the presented results, we can state that macrolithic artifacts are a suitable tool for testing the settlement structure of preliterate societies.

## Supporting information

S1 Video Aerial view of Güvercinkayası site by Ü. Çakir and A. Hakkut 2015. GK archive.
(MP4)

S1 Fig General plan of the Güvercinkayası site, excavated according to a grid system 1996-2017. A: In red: Sporadic remains of architecture from the last, Late Chalcolithic level (Level III; 4,700 – 4,400 cal BCE); in green and yellow: Middle Chalcolithic levels (Levels I-II; 5,200 – 4,820/4,750 cal BCE). B: Geomorphological plan of the terrain after excavation. The Melendiz River skirts its western edge. Maps by D. Pilař.
(TIF)

S2 Fig 3D models of artifacts. https://skfb.ly/prGut. Fig 17: 1 – U-type roughout GK10_218; Fig. 17: 2 – U-type roughout GK11_256; Fig 17: 3 – U-type roughout GK03_82; Fig 18: 1 - L1-subtype GK07_131; Fig 18: 2 - L2-subtype GK09_131; Fig 18: 3 - L2-subtype GK11_38; Fig 18: 4 - L2-subtype GK11_329); Fig 18: 5 - L2-subtype GK11_41; Fig 19: 1 - L3-subtype GK02_70; Fig 19: 2 - L3-subtype GK11_258; Fig 19: 3 - L4-subtype with modelled “hand” on dorsal side GK01_18; Fig 20: 1 - U3-subtype with two utilization areas GK10_71; Fig 20: 2 - U3-subtype GK03_44; Fig 20: 3 - U4-subtype with two utilization areas GK11_190; Fig 21: 1 - LM-subtype GK10_67; Fig 21: 2 - M1-subtype GK08_157; Fig. 21: 3 - M2-subtype GK08_48; Fig 35: 1 – T-class GK09_36; Fig 35: 2 – T-class GK10_207. The abbreviation GK is the designation of the site, the following two digits indicate the year of the excavation, the last two digits are the inventory number of the find. Author D. Pilař.
(DOCX)

S1 Text Detailed classification system used in the paper. For comparison, see Figs 8-14.
(DOCX)

S1 Dataset Database of all macrolithic artifacts in the assemblage. YEAR: Season of excavation. INVENTORY: Inventory number given by excavators. FIND: Find number within the unit. NEW NUMBER: Given number in case of missing inventory number. GIS_XY: Localization in the grid system used for excavation and documentation (see S1). SETTLEMENT: Localization in the Lower or Upper settlement, if possible (see Fig 1). GROUP: Localization in the smaller units on the site, if possible (see Fig 3). PRESERVATION: 1: Complete artifact; 2: Fragment. %: An estimate of the percent preservation of the artifact. NUMBER OF FACES: Number of utilization areas. CLASS/TYPE: Main class or type in the case of GMT (see Table 1 and Fig 6). OUTLINE: Class and type dependent (see Figs 8–11, 13, 14). FACE: Position of utilization areas for L, U, M types of GMT-class (see Figs 8, 9). WORK PART: Position of the use traces for PRT and PS (Figs 11, 13). WORK TRACES: Macroscopically visible use traces on PRT and A (see Figs 12, 13). LS: Outline of longitudinal section (see Figs 8–10, 14). TS: Outline of transverse section (see Figs 8, 9, 12). HANDLES: Position of ergonomic features of U-types on dorsal part (see Fig 9). SUBTYPE: Morphometric subtypes of some classes. SECONDARY: Secondary use of artifacts. MANUFACTURE: Method of manufacture. LENGTH: Maximum length in centimeters. WIDTH: Maximum width in centimeters. THICKNESS: Maximum thickness in centimeters. WEIGHT: Weight in kilograms. NOTE: Metrics of special parts (see Fig 7), and note if needed (e.g., presence of the striking points). MATERIAL: Raw material determination. STRUCTURE: Structure of some raw materials, typically basalts.
(XLSX)

S1 Table Raw materials of the classes and types. For abbreviations see Table 1. Author K. Doležalová.
(XLSX)

S2 Table Table of type representation in Lower and Upper settlement. For abbreviations see Table 1. Author D. Pilař.
(XLSX)

S3 Table Table of subtype representation in Lower and Upper settlement. For abbreviations see text. Author D. Pilař.
(XLSX)

S4 Table Comparison of metric properties of individual classes and types in Lower and Upper settlement. **Tests of statistical significance, in case of representative groups.** Author D. Pilař.
(XLSX)

S5 Table Comparison of metric properties of individual subtypes in Lower and Upper settlement. **Tests of statistical significance, in case of representative groups.** Author D. Pilař.
(XLSX)

S6 Table Table of class and type representation in spatial units. Author D. Pilař.
(XLSX)

S7 Table Table of class and type representation in selected representative spatial units. Author D. Pilař.
(XLSX)

S8 Table PCA of class and type representation in selected representative spatial units. Author D. Pilař.
(XLSX)

S9 Table Comparison of metric properties of individual classes and types in spatial units. **Tests of statistical significance, in case of representative groups.** Author D. Pilař.
(XLSX)

S10 Table Table of subtype representation in spatial units. Author D. Pilař.
(XLSX)

S11 Table PCA of L-subtypes representation in selected representative spatial units. Author D. Pilař.
(XLSX)

S12 Table PCA of PRT-subtypes representation in selected representative spatial units. Author D. Pilař.
(XLSX)
