# Supplementary figures and images for: Behind the wall: Macrolithic artifacts as testing tools for activities and social structure on a Middle Chalcolithic site in Central Anatolia
Source: PLoS One. 2025 Apr 14;20(4):e0319698. doi: 10.1371/journal.pone.0319698 (PMC11996077; doi:10.1371/journal.pone.0319698)

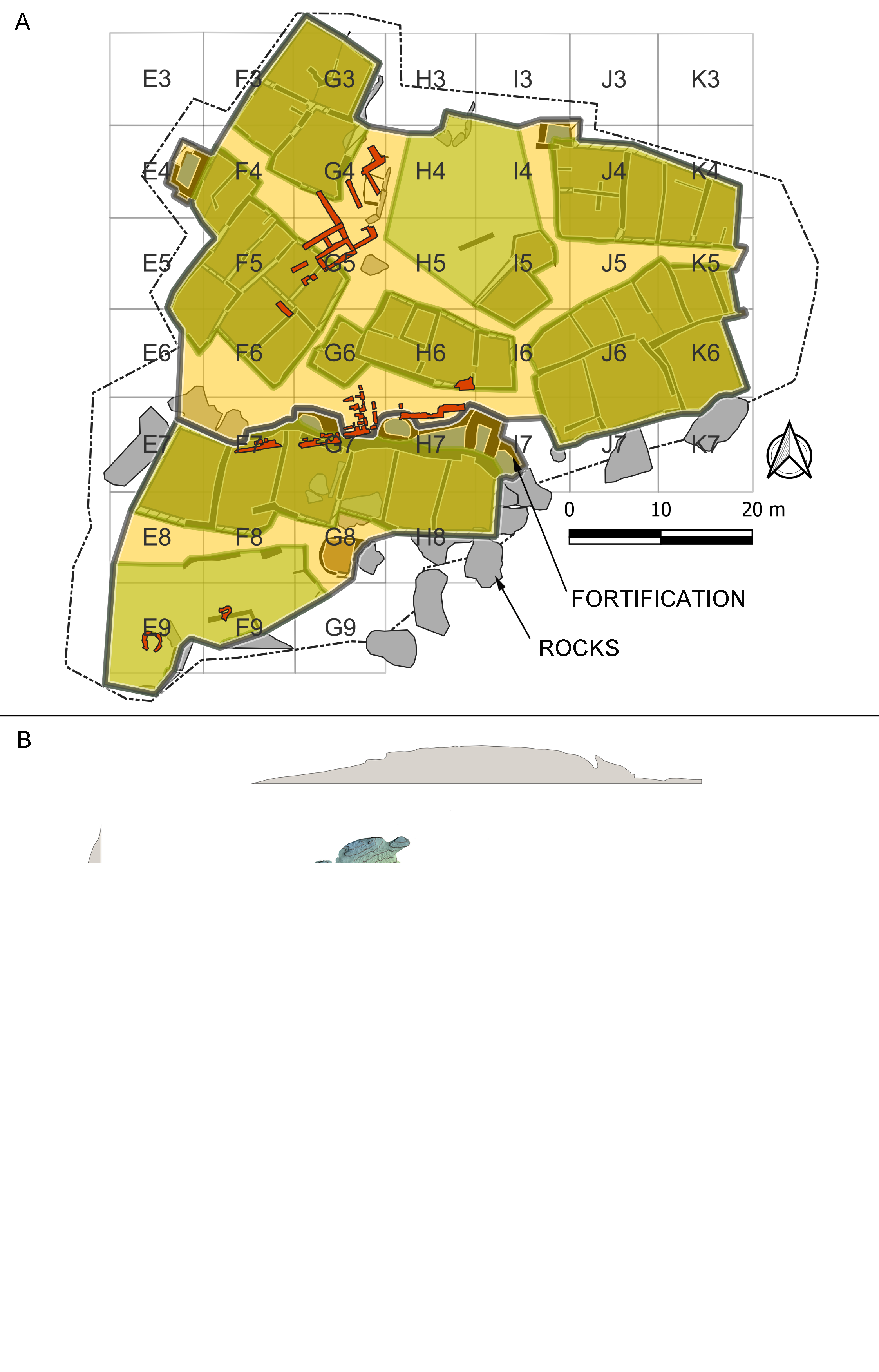

Supplement: S1 Fig — A: In red: Sporadic remains of architecture from the last, Late Chalcolithic level (Level III; 4,700 – 4,400 cal BCE); in green and yellow: Middle Chalcolithic levels (Levels I-II; 5,200 – 4,820/4,750 cal BCE). B: Geomorphological plan of the terrain after excavation. The Melendiz River skirts its western edge. Maps by D. Pilař. (TIF) [file pone.0319698.s002.tif]
