# Supplementary material for: Behind the wall: Macrolithic artifacts as testing tools for activities and social structure on a Middle Chalcolithic site in Central Anatolia
Source: PLoS One. 2025 Apr 14;20(4):e0319698. doi: 10.1371/journal.pone.0319698 (PMC11996077; doi:10.1371/journal.pone.0319698)
Supplement: S2 Fig — https://skfb.ly/prGut. Fig 17: 1 – U-type roughout GK10_218; Fig. 17: 2 – U-type roughout GK11_256; Fig 17: 3 – U-type roughout GK03_82; Fig 18: 1 - L1-subtype GK07_131; Fig 18: 2 - L2-subtype GK09_131; Fig 18: 3 - L2-subtype GK11_38; Fig 18: 4 - L2-subtype GK11_329); Fig 18: 5 - L2-subtype GK11_41; Fig 19: 1 - L3-subtype GK02_70; Fig 19: 2 - L3-subtype GK11_258; Fig 19: 3 - L4-subtype with modelled “hand” on dorsal side GK01_18; Fig 20: 1 - U3-subtype with two utilization areas GK10_71; Fig 20: 2 - U3-subtype GK03_44; Fig 20: 3 - U4-subtype with two utilization areas GK11_190; Fig 21: 1 - LM-subtype GK10_67; Fig 21: 2 - M1-subtype GK08_157; Fig. 21: 3 - M2-subtype GK08_48; Fig 35: 1 – T-class GK09_36; Fig 35: 2 – T-class GK10_207. The abbreviation GK is the designation of the site, the following two digits indicate the year of the excavation, the last two digits are the inventory number of the find. Author D. Pilař. (DOCX) [file pone.0319698.s017.docx]

These 3D figures are stored here: https://skfb.ly/prGut
