# Supplementary material for: Behind the wall: Macrolithic artifacts as testing tools for activities and social structure on a Middle Chalcolithic site in Central Anatolia
Source: PLoS One. 2025 Apr 14;20(4):e0319698. doi: 10.1371/journal.pone.0319698 (PMC11996077; doi:10.1371/journal.pone.0319698)
Supplement: S1 Text — Detailed classification system used in the paper. For comparison, see Figs 8-14. (DOCX) [file pone.0319698.s003.docx]

**DETAILED CLASSIFICATION SYSTEM**

See Figs 8–14 in the text

**CLASS: GRINDING-MILLING TOOLS (GMT)**

**TYPE: LOWER STONES (L)**

These are passive tools, part of lower-upper stone sets, reaching about 50 cm in length and 30 cm in width. L-types can be classified (Fig 8) according to the overall shape when looking at the working part (O), according to the position of the active surface (F), according to the longitudinal section (LS) and transversal section (TS). In some cases, it is possible to distinguish a LOWER STONE and MORTAR that were used in combination (LM), or an UPPER STONE and LOWER STONE that were used in combination (UL). The method of manufacture of L-types (coarse flaked, flaked, ground) and shaping of their base (flat, irregular) was also observed. Roughouts in various stages of production were collectively marked RL.

**TYPE: UPPER STONES (U)**

U-types measure up to 30 cm in length and 20 cm in width, and are an active tool of the lower-upper stone sets. U-types can be divided into six shapes when looking at the working part (O) (Fig 9). Also for these types, the tools can best be identified by the curvature of the longitudinal (LS) and transverse (TS) sections. Furthermore, the occurrence and position of so-called ergonomic features, sometimes termed as handles (H), were monitored; these are usually located either on one of the shorter sides (H1; H2) or on both sides (H3). Also in the case of U-types, the method of manufacture (coarse flaked, flaked, ground) was recorded. Roughouts in various stages of production are collectively marked RU.

**TYPE: MORTARS (M)**

Mortars are passive tools with massive walls, measuring up to about 30 cm in length and 30 cm in width. M-types could best be distinguished by their shapes when looking at the working part (O; Fig 10), and further by the regularly rounded active surface (F), with regular concave levelling. M-types differ in terms of the position of the active surface (F1 is in the center of the tool, F2 is located asymmetrically) and according to the shape of the longitudinal section (LS). Roughouts in various stages of production are collectively labeled RM.

**TYPE: PESTLES (PS)**

These active tools should be compatible with mortars. PS-types in the GK assemblage are oblong in shape, with an oval cross-section and reach a length of about 20 cm and a width of up to 10 cm. In the GK assemblage (Fig 11) they are distinguished according to their shape in plan view (O) and according to the shape of the working part (WP) in plan view.

**CLASS: VESSELS (V)**

Items in this class have a maximum length of about 20 cm and a width of 15-20 cm. They include deep bowl-shaped vessels with thin walls. In the vast majority of cases they are made from softer raw materials although there are some exceptions to this rule.

​

**CLASS: ABRADERS (A)**

This class includes items that bear various abraded surfaces, or very typical straight U-shaped grooves, and they are usually made of materials suitable for reducing various materials. Abraders in the GK assemblage usually reach a length of about 30 cm and a width of about 20 cm. In this work, we distinguish them (Fig 12) on the basis of their transverse section (TS), and according to evidence of use traces, such as abraded flat surfaces (WT1) and straight grooves (WT2). However, items from other classes, such as L-types, and U-types, as well as T-class, PRT-class and D-class may also have been secondarily used as abraders because they were made from a suitable material.

**CLASS: MULTIPLE-USE TOOLS (PRT)**

This quantitatively rich and morphologically broad class of artifacts, with a maximum length of about 15 cm and a width of about 10 cm, usually did not require any complex shaping (pebbles, cobbles, suitable pieces of eroded rocks). They can be classified (Fig 13) according to several criteria: Shape in elevation (O), and also according to the location of macroscopically visible traces of use (WP) and their type – polished (WT1), pounding/fine hammering (WT2), coarse hammering (WT3), striations (WT4), dimples (WT5). In our classification system, we do not divide this class into types in the first step of analyses (polishers, percussion tools, etc.), because most often these are multifunctional tools bearing multiple traces of use, in different utilization areas. The visibility of some macroscopic use-wear depends on the raw materials. This broad group of tools deserves deeper experimental comparative analyses, but these are beyond the scope of this work.

**CLASS: DRILLED ITEMS (D)**

The artifacts that constitute this class may be round, oval or sometimes irregular in shape and have a maximum length of 15 - 20 cm and a width of 15 - 20 cm; their essential distinguishing is a perforation, probably produced by drilling. They can be distinguished (Fig 14) according to their shape in the plan view (O) and according to the longitudinal section (LS). Semi-finished products (roughouts), mostly cracked during drilling (RD), are also recorded here.

**CLASS: STONE SPHERES (B)**

This is a very specific class made up of almost regular stone spheres, which have a maximum diameter of 2 cm to 10 cm.

**CLASS: PLATES WITH DIMPLES (T)**

These are variously shaped plate formed pieces of rock, mostly of oval shape, less often of irregular shape, bearing a number of spatially organized dimples. Dimples are always placed on one face of the artifact only.

**CLASS: DOOR SOCKETS (S)**

These are vary in shape and are mostly made from local raw materials. The occurrence of holes with different diameters and irregularly shaped transverse-sections is significant. The bottom of the irregularly concave active surface is usually flat.

**CLASS: AXES/ADZES (AXE)**

This class of tools, originally with cutting edges, is rare in the GK assemblage. We recorded only two pieces with a preserved cutting edge. They were mostly secondarily used as hammer stones.

**CLASS: OTHERS**

A group of rare finds. For example, variously shaped stones of local ignimbrite, apparently serving as binding stones, are included here.
